# Supplementary material for: DsbA-L mediated renal tubulointerstitial fibrosis in UUO mice
Source: Nat Commun. 2020 Sep 18;11:4467. doi: 10.1038/s41467-020-18304-z (PMC7501299; doi:10.1038/s41467-020-18304-z)
Supplement: Supplementary file 1 — Supplementary Information [file 41467_2020_18304_MOESM1_ESM.docx]

**DsbA-L mediated renal tubulointerstitial fibrosis in UUO mice**

XiaozhouLi, Jian Pan, Huiling Li, Guangdi Li, Xiangfeng Liu, Bohao, Liu, Zhibiao He, ZhengyuPeng, HongliangZhang,Yijian Li, Xudong Xiang, Xiangping Chai, YunchangYuan，Peiling Zheng，Feng Liu, Dongshan Zhang

(A) Comparison of DsbALsequencesin human and mouse


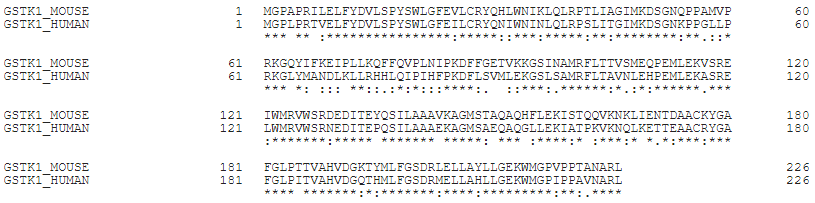


(B) Comparison of HSP90 sequences in human and mouse


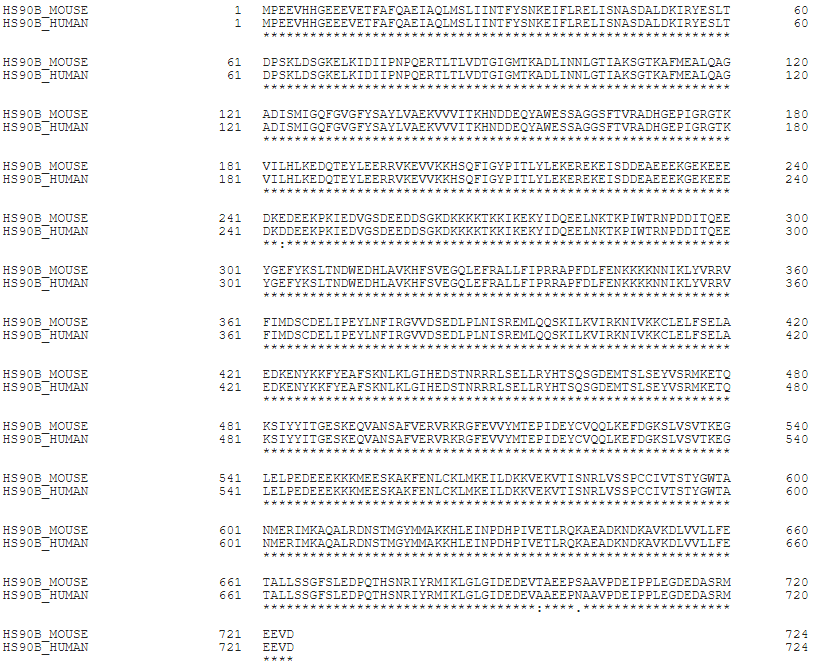


**Supplementary Figure 1**. Sequence similarity of DsbAL(A) and HSP90 (B) in human and mouse. Sequences of or GSTK1 were extracted from the UniProt database with the following ID information: DsbAL in human (Q9Y2Q3), DsbAL in mouse (Q9DCM2), HSP90 in human (P08238), and HSP90 in mouse (P11499). Figures were produced by sequence online tools at <https://www.uniprot.org/align>.

Among 226 amino acid positions of DsbAL, 161 positions are identical and 51 positions are similar between human and mouse sequences. Only 15 (6.6%) amino acid positions showed a different pattern.

Among 724 amino acid positions of HSP90, 721 positions are identical and 3 positions are similar between human and mouse sequences. There is no (0%) amino acid position with a different pattern.

**
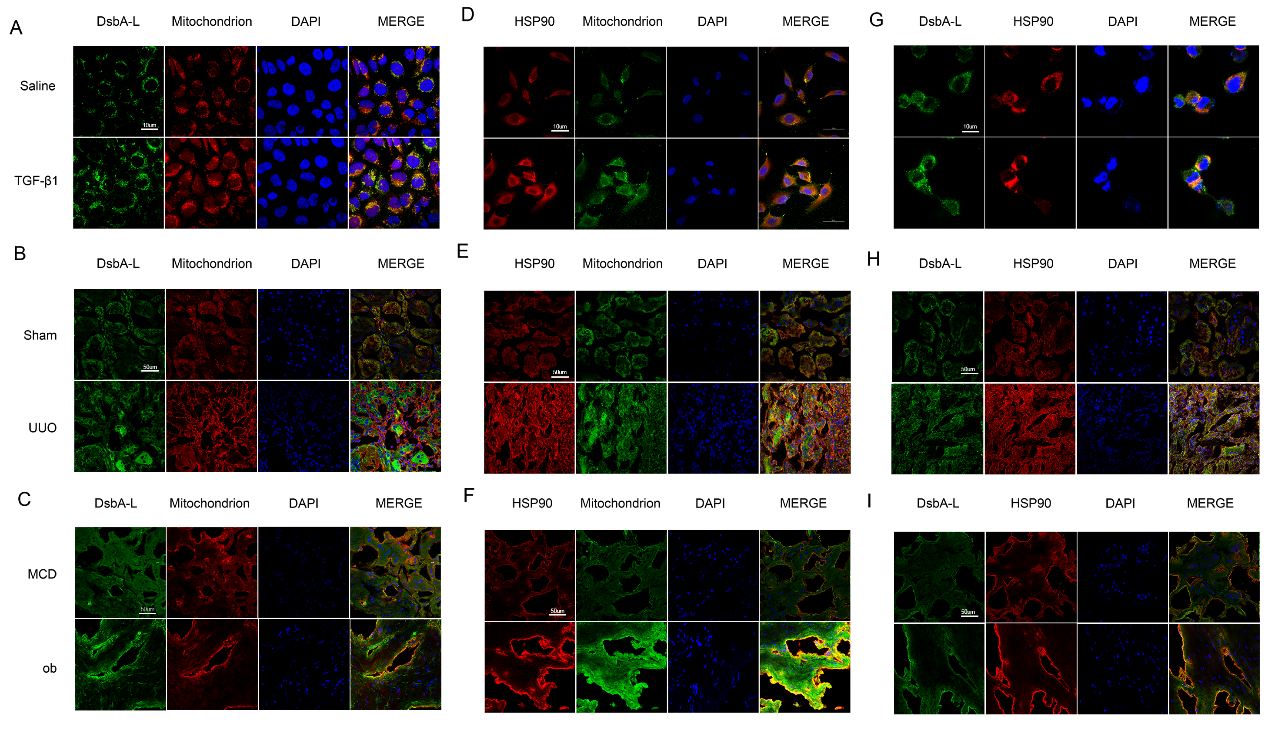
**

**Supplementary Figure 2**:**Colocalization of DsbA-L and HSP90 in the mitochondria of BUMPT cells and kidneys of UUO mice and Ob patients.**(A-C) Localization of DsbA-L in the mitochondria of BUMPT cells treatment with or without TGF-β1and the kidneys of mice model in the sham and UUO groups as well as MCD and Ob patients. (D-F) Localization of HSP90 in the mitochondria of BUMPT cells treated with or without TGF-β1 and the kidneys of sham and UUOmice as well as MCD and Ob patients. (J-I) Colocalization of DsbA-L and HSP90 in BUMPT cells treated with or without TGF-β1 and the kidneys of sham and UUO mice as well as MCD and Ob patients. Each experiment was repeated 6 times independently with similar results.

###
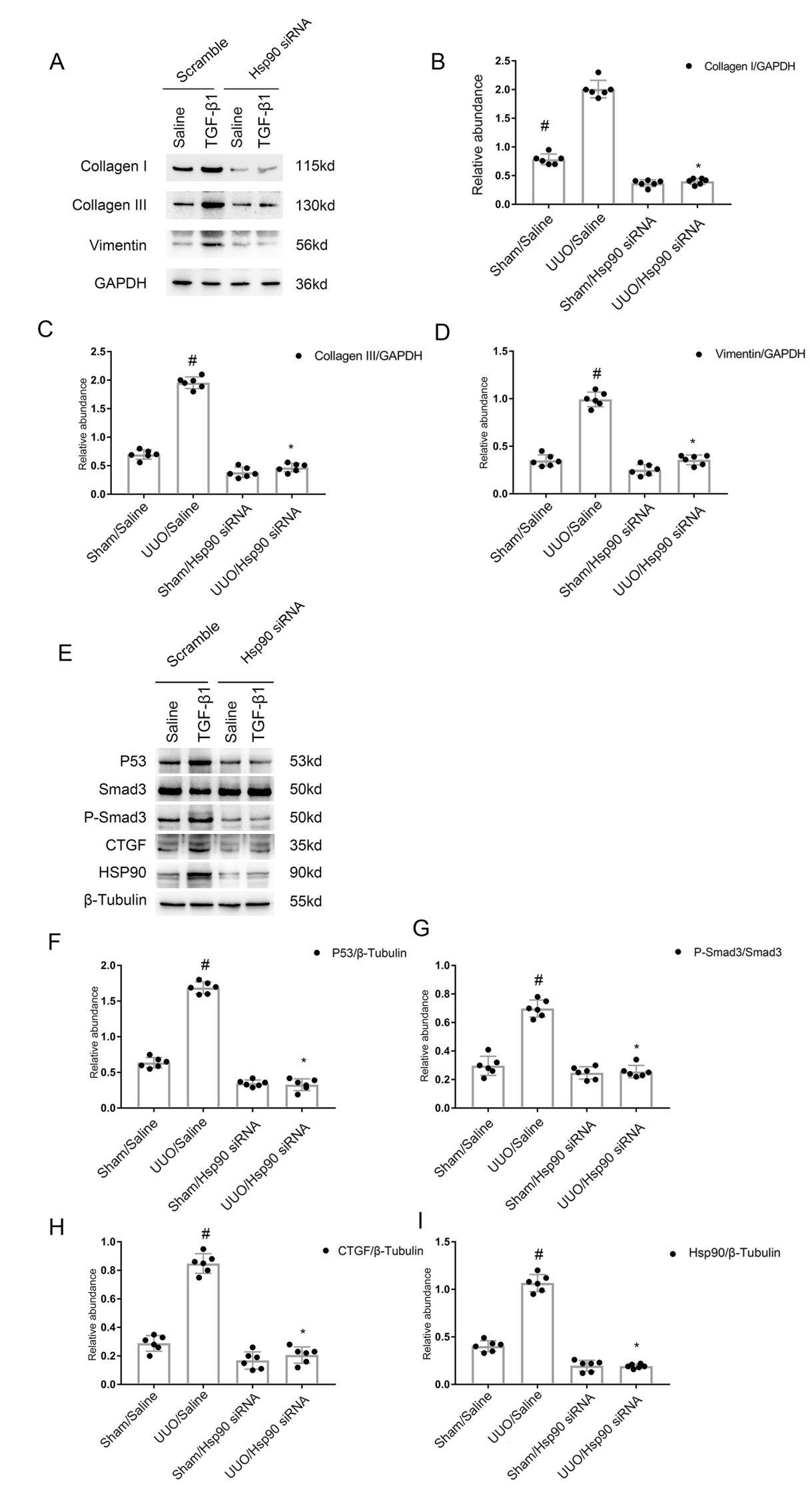


**Supplementary Figure 3: Inhibition of HSP90 by** Hsp90β siRNA **attenuated the accumulation of ECM via inactivation of Smad3 and down-regulation of p53 and CTGF.**The Hsp90β siRNA was transfected into BUMPT cells and treated with or without 5ng/ml TGF-β1 for 24hours. (A&E) Immunoblot analysis of Col 1&III, ɑ-SMA, TGF-β1, CTGF, DsbA-L, p-Smad3 and p53. Each experiment was repeated 6 times independently with similar results. (B-D&F-I)Analysis of the grayscale image between them. Data are expressed as means ± sd(n=6). # *P<0.05* versus saline group. **P<0.05* versus TGF-β1 group. (B-D&F-I) indicate the statistical Student's T test used(means ± sd,n=6,P<0.05) .


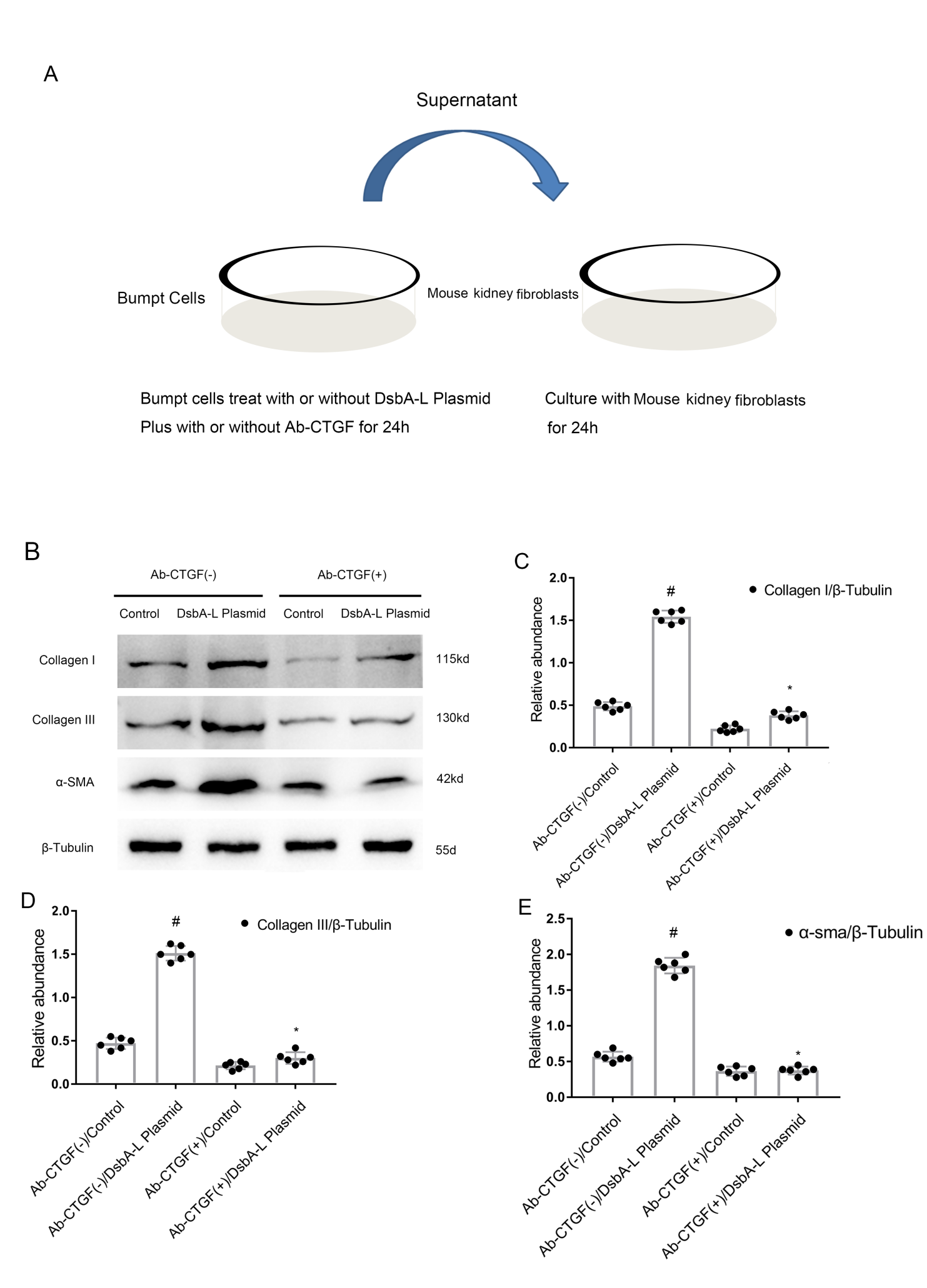


**Supplementary Figure 4: Co-culture of** **mouse kidney fibroblasts and BUMPT cells transfected with DsbA-Lleaded to the accumulation of ECM in mouse kidney fibroblasts via secretion of CTGF**. The supernatant of BUMPT cells transfected with the plasmid of DsbA-L plus with or without CTGF neutralizing antibody treated mouse kidney fibroblasts for 24hours. (A) The Co-culture model of mouse kidney fibroblasts and BUMPT cells. (B)Immunoblot analysis of Col 1&III, and ɑ-SMA. (B)Each experiment was repeated 6 times independently with similar results. (C-E) Analysis of the grayscale image between them. Data are expressed as means ± sd(n=6). # *P<0.05* versus the control group. **P<0.05* versus DsbA-L group. (C-E) indicate the statistical Student's T test used(means ± sd,n=6,P<0.05).


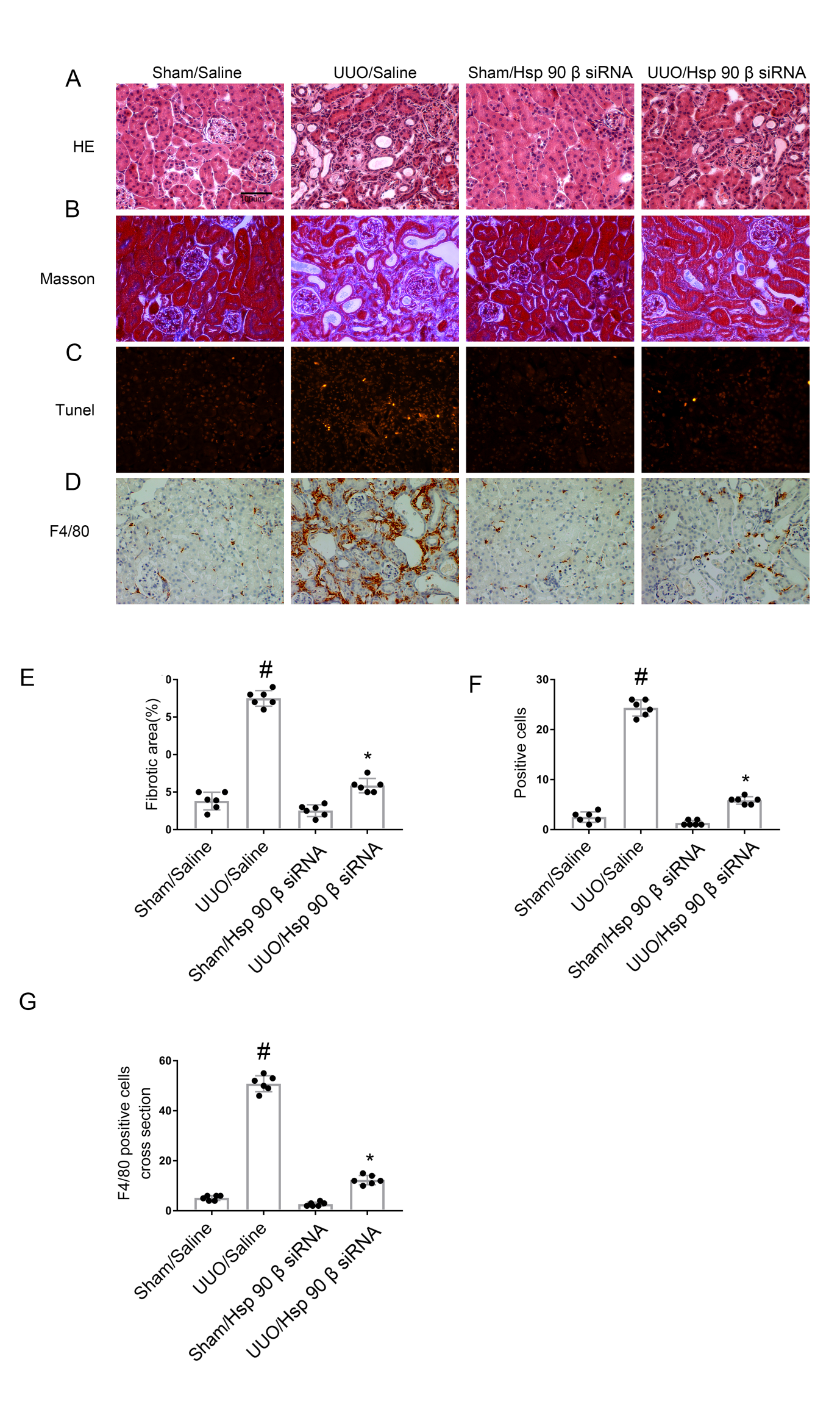


**Supplementary Figure 5: Amelioration of UUO-induced renal fibrosis and renal cell apoptosis in mice treated withHsp90β siRNA.** Male C57BL/6 mice were subjected to UUO and then treated with or without 15mg/kgHsp90β siRNA twice a week. (A) Representative Hematoxylin and eosin staining. (B) Representative Masson trichrome staining. (C)Representative sections of TUNEL-positive cells. (D)Representative sections of F4/80-positive cells. (A-D)Each experiment was repeated 6 times independently with similar results.(E) Quantification of tubulointerstitial fibrosis in the kidney cortex. (F) The number of TUNEL-positive cells. (G) The number of F4/80-positive cells.Original magnification x 400. Scar bar:100um. Data are expressed as means ± sd(n=6). # *P<0.05* versus sham group. * *P<0.05* versus UUO group. (E-G) indicate the statistical Student's T test used(means ± sd,n=6,P<0.05).

**
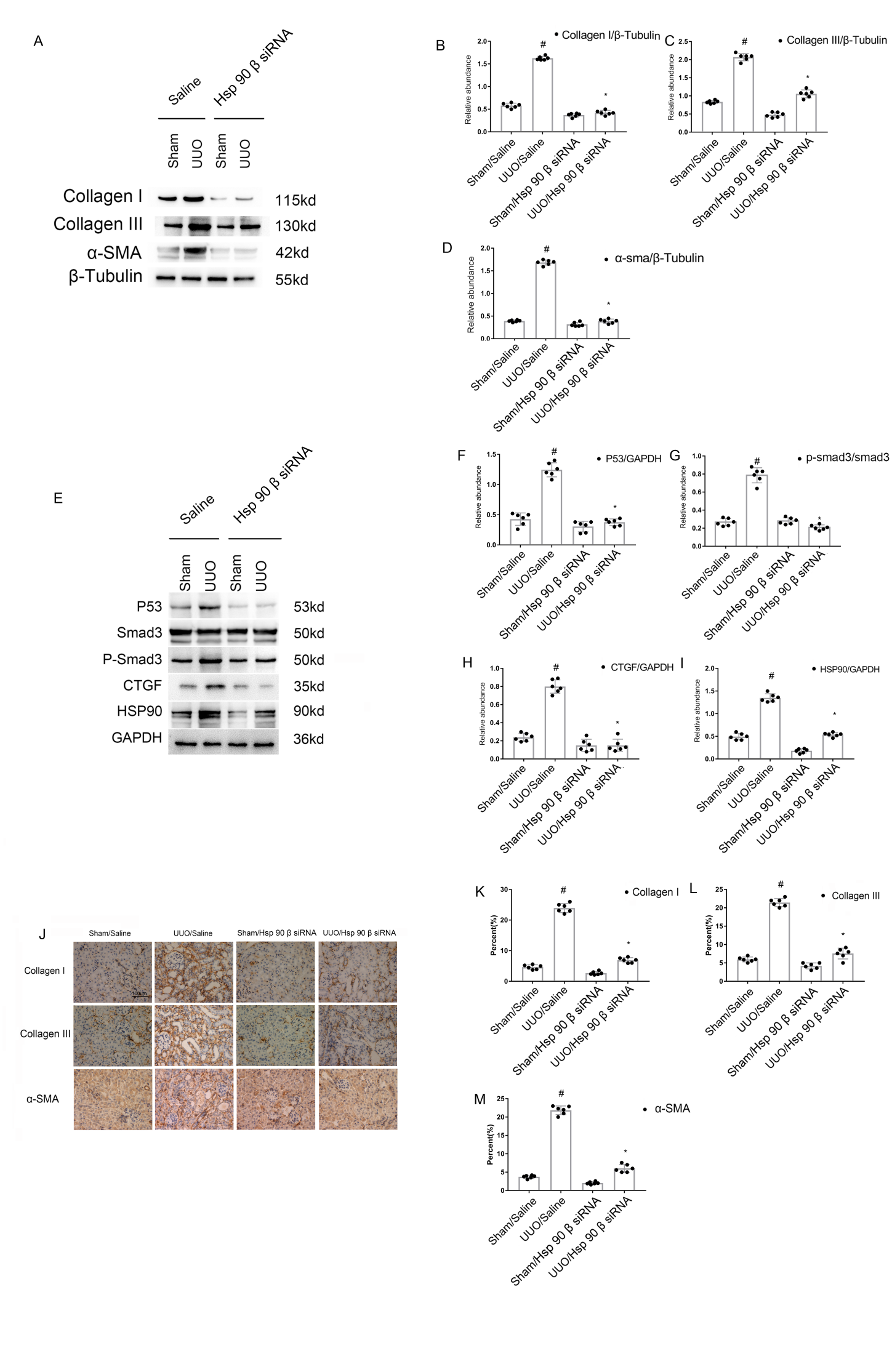
**

**Supplementary Figure 6: Attenuation of UUO-induced** **expression of Col 1&III, ɑ-SMA, CTGF, p-Smad3 and p53 inmicetreatedwith****HSP90 siRNA.** Male C57BL/6 mice were subjected to UUO and treated with or without 15mg/kg Hsp90β siRNA twice a week.(A&E) Immunoblot analysis of Col 1&III, ɑ-SMA, CTGF, p-Smad3 and p53. (B-D&F-I) Analysis of the grayscale image between them. (J) Immunohistochemical staining of Col 1&III and ɑ-SMA. (K-M) Quantification of immunohistochemical staining. (A,E&J)Each experiment was repeated 6 times independently with similar results.Original magnification x 400. Scar bar:100um. Data are expressed as means ± sd(n=6). # *P<0.05* versus sham group. * *P<0.05* versus UUO group. （B-D,F-I&K-M）indicate the statistical Student's T test used(means ± sd,n=6,P<0.05) .


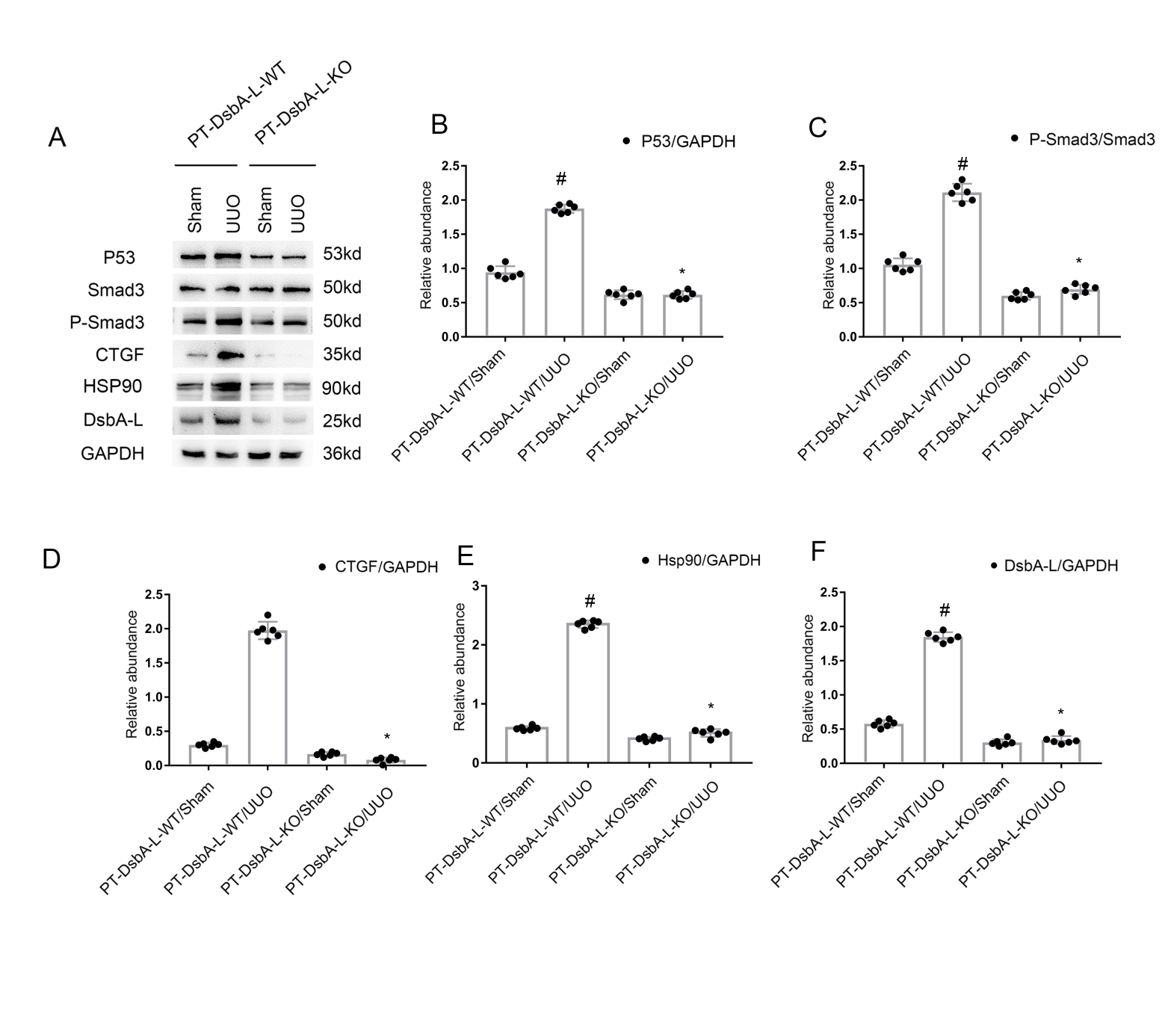


**Supplementary Figure 7:****UUO-induced expression of HSP90, CTGF, p-Smad3 and p53 were reduced in PT-DsbA-L-KO mice.** The left ureter of PT-DsbA-L-KO and PT-DsbA-L-WT littermate mice was ligated for seven days to establish a UUO model. (A) Immunoblot analysis of HSP90, CTGF, p-Smad3 and p53. (A)Each experiment was repeated 6 times independently with similar results.(B-F) Analysis of the grayscale image between them. Data are expressed as means ± sd(n=6). # *P<0.05* versus sham group. * *P<0.05* versus PT-DsbA-L-WT with UUO group. (B-F) indicate the statistical Student's T test used(means ± sd,n=6,P<0.05) .


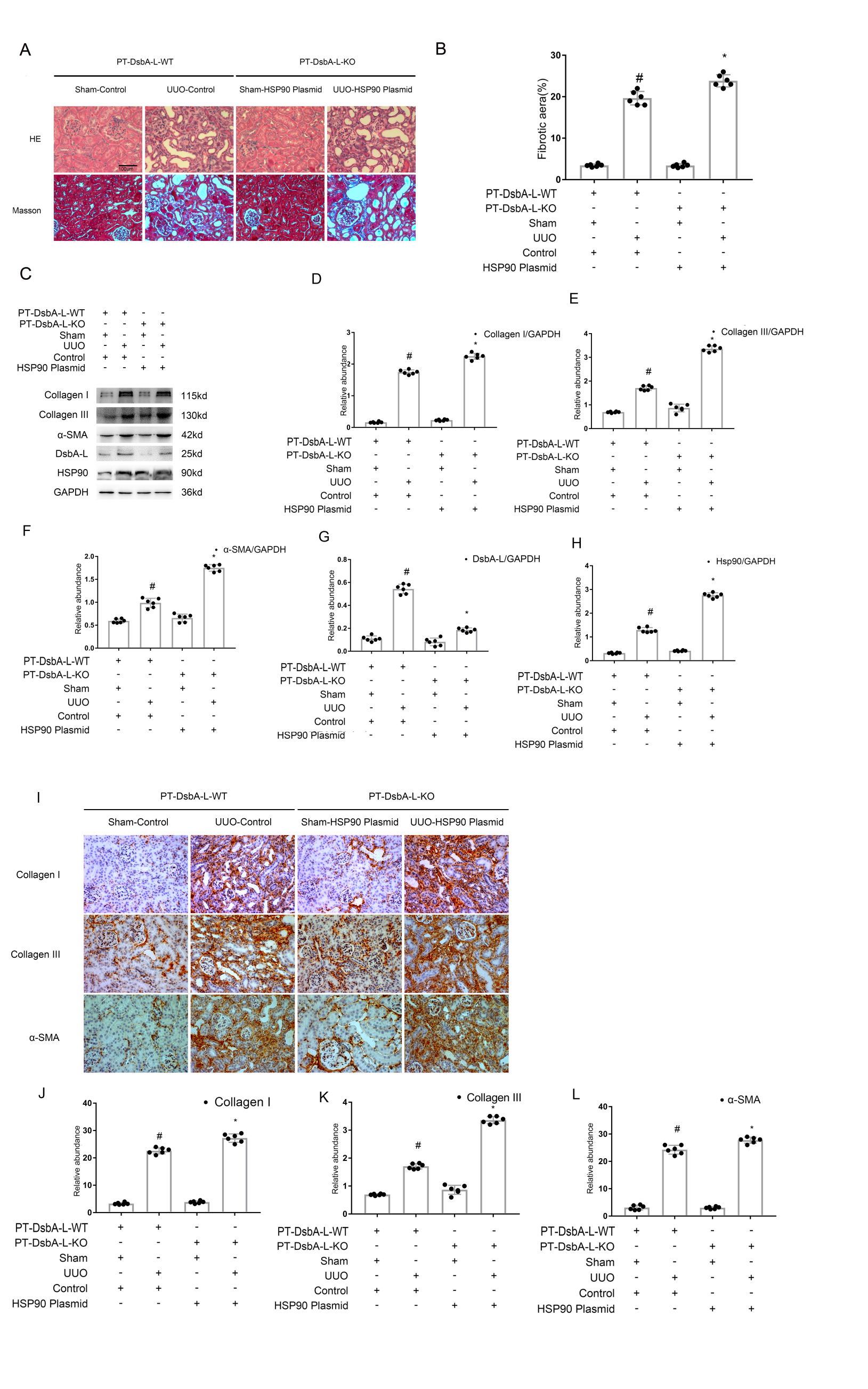


**Supplementary Figure 8:** **PT-DsbA-L-KO attenuated the UUO-induced renal fibrosis in mice and was diminished by the overexpression of** **HSP90.**The left ureter of PT-DsbA-L-KO and PT-DsbA-L-WT littermate mice was ligated to establish the UUO model and then treated with or without HSP90 plasmid for seven days. (A) Representative the staining of Hematoxylin and eosin and Masson trichrome. (B) Quantification of the tubulointerstitial fibrosis in the kidney cortex. (C)Immunoblot analysis of Col 1&III, ɑ-SMA, DsbA-L, HSP90and β-tubulin. (D-H)Analysis of the grayscale image between them.(I) Immunohistochemical staining of Col 1&III and ɑ-SMA. (A,C&I)Each experiment was repeated 6 times independently with similar results. (J-L) Quantification of immunohistochemical staining. Original magnification x 400.Scar bar:100um.Data are expressed as means ± sd (n=6). # P<0.05 versus sham group. * P<0.05 versus UUO group. (B,D-H&J-L) indicate the statistical Student's T test used(means ± sd,n=6,P<0.05) .


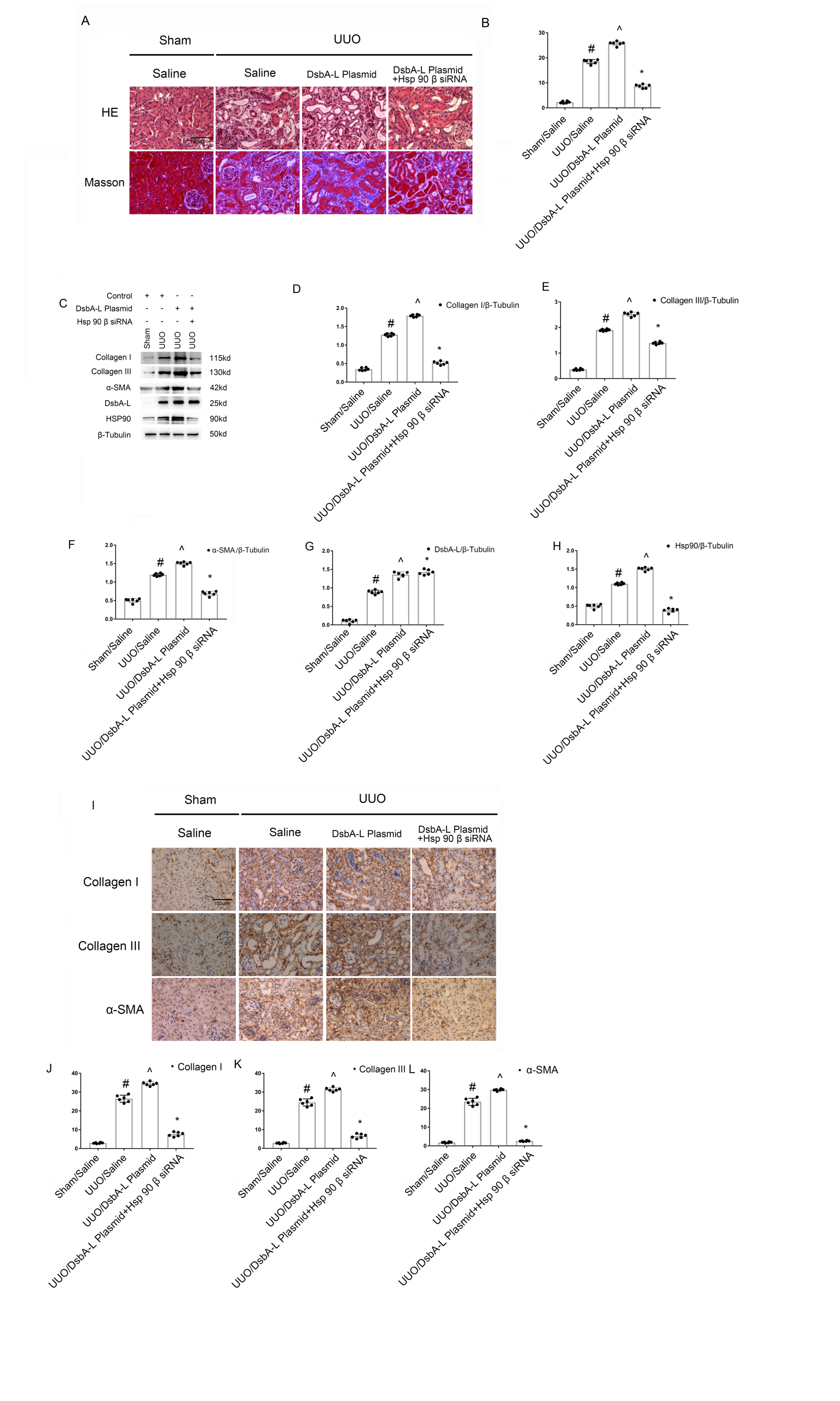


**Supplementary Figure 9: UUO-induced renal fibrosis was enhanced by overexpression of DsbA-L except in UUO mice treated with Hsp90β siRNA.** Male C57BL/6 mice were subjected to UUO and then treated with 15mg/kg/HSP90 siRNA twice a week plus DsbA-L once a week for seven days. (A) Representative the staining of Hematoxylin and eosin and Masson trichrome. (B) Quantification of the tubulointerstitial fibrosis in the kidney cortex.(C) Immunoblot analysis of Col 1&III, ɑ-SMA, DsbA-L, HSP90 and β-tubulin. (A,C&I)Each experiment was repeated 6 times independently with similar results. (D-H)Analysis of the grayscale image between them. (I) Immunohistochemical staining of Col 1&III and ɑ-SMA. (J-L) Quantification of immunohistochemical staining.Original magnification x 400. Scar bar:100um. Data are expressed as means ± sd(n=6).#*P<0.05* versus sham group.ˆ or * *P<0.05* versus UUO group. (B,D-H&J-L) indicate the statistical Student's T test used(means ± sd,n=6,P<0.05) .


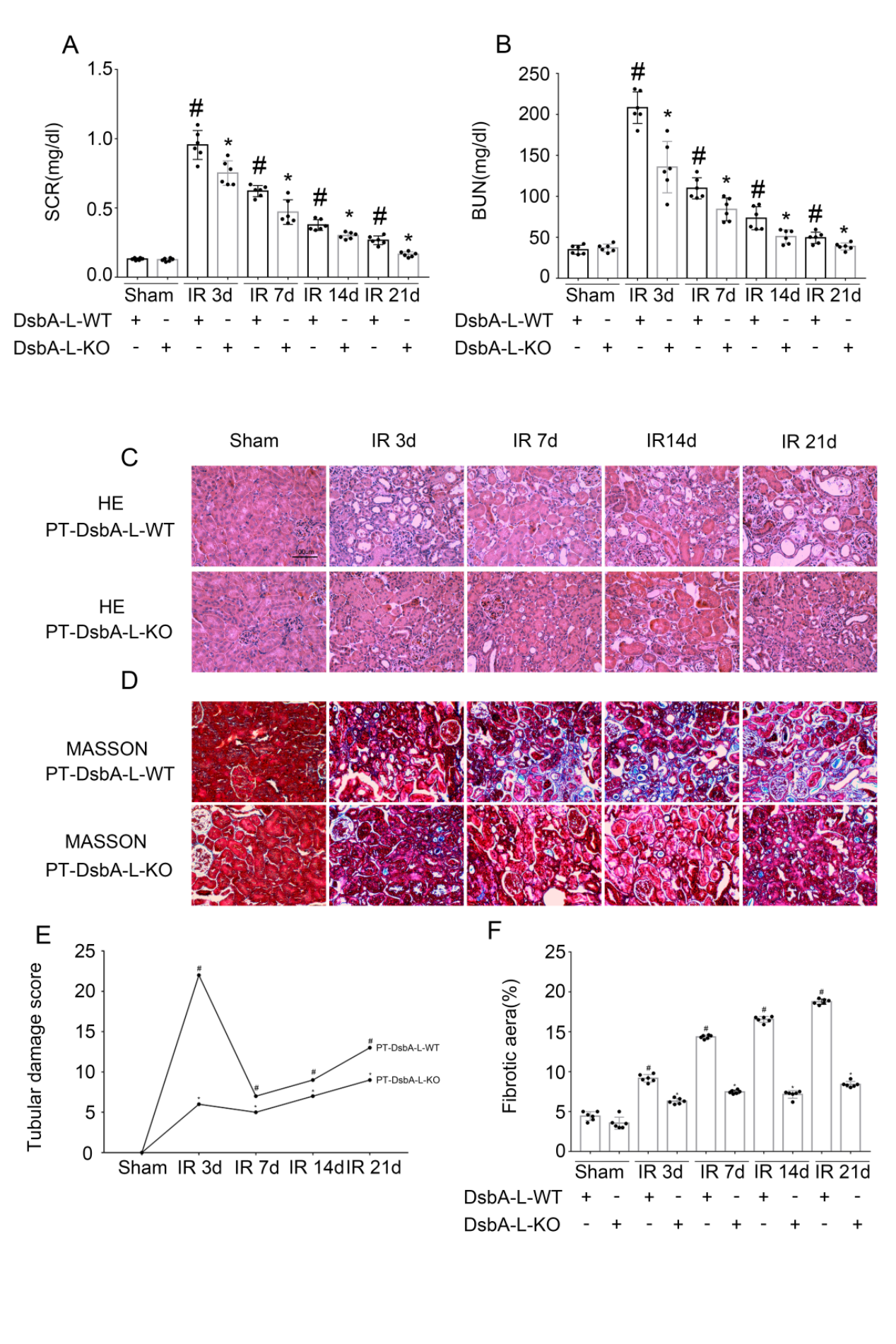


**Supplementary Figure 10**:**PT-DsbA-L-KO attenuated the I/R-induced renal fibrosis in mice.** The PT-DsbA-L-KO and PT-DsbA-L-WT littermate mice were subjected to 28 minutes of bilateral renal ischemia followed by 3–21 days of reperfusion. The blood samples were collected to measure the BUN(A) and Serum creatinine(B). (C) Representative Hematoxylin and eosin staining. (D)RepresentativetheMasson trichrome staining. (E) Representative the tubular damage scores. (F) Quantification of the tubulointerstitial fibrosis in the kidney cortex.These data are representative of at least four separate experiments shown as means±sd (n=6).#*P<0.05* versus the sham group. * *P<0.05* versus I/R group. (C&D)Each experiment was repeated 6 times independently with similar results. (A-B&E-F) indicate the statistical Student's T test used(means ± sd,n=6,P<0.05) and Original magnification, x400. Scar bar:100um


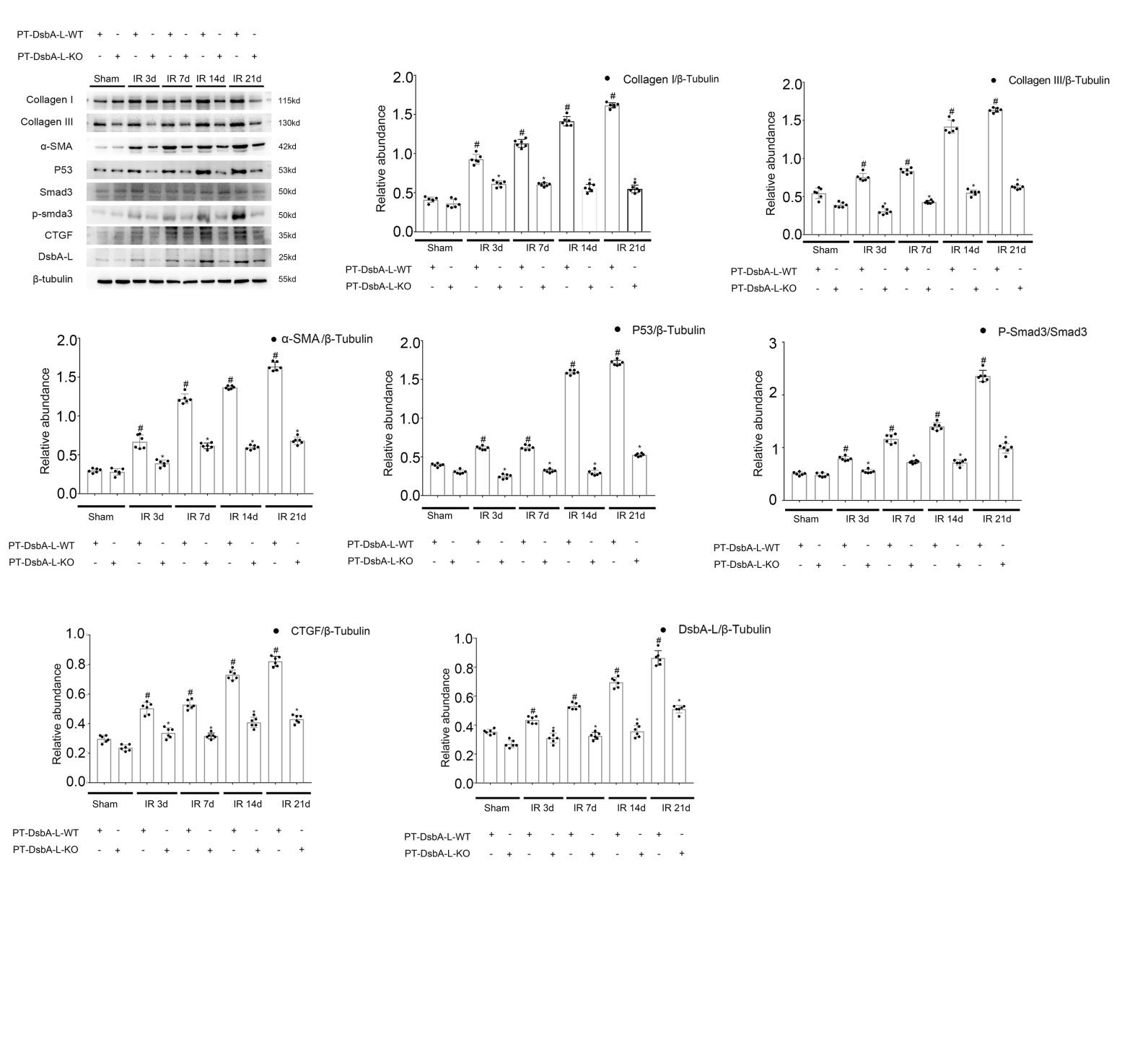


**Supplementary Figure 11**:**PT-DsbA-L-KO attenuated the I/R-induced** **DsbA-L/HSP90/p53 and Smad3/CTGF/ ECM axis in mice.** The PT-DsbA-L-KO and PT-DsbA-L-WT littermate mice were subjected to 28 minutes of bilateral renal ischemia followed by 3–21 days of reperfusion. (A) Immunoblot analysis of Col 1&III, ɑ-SMA, CTGF, p53, Smad3, p-Smad3, CTGF, HSP90, and β-tubulin. (A)Each experiment was repeated 6 times independently with similar results. (B-H) Analysis of the grayscale image between them.These data are representative of at least four separate experiments shown as means±sd (n=6). # *P<0.05* versus sham group. * *P<0.05* versus I/R group. (B-H) indicate the statistical Student's T test used(means ± sd,n=6,P<0.05) .


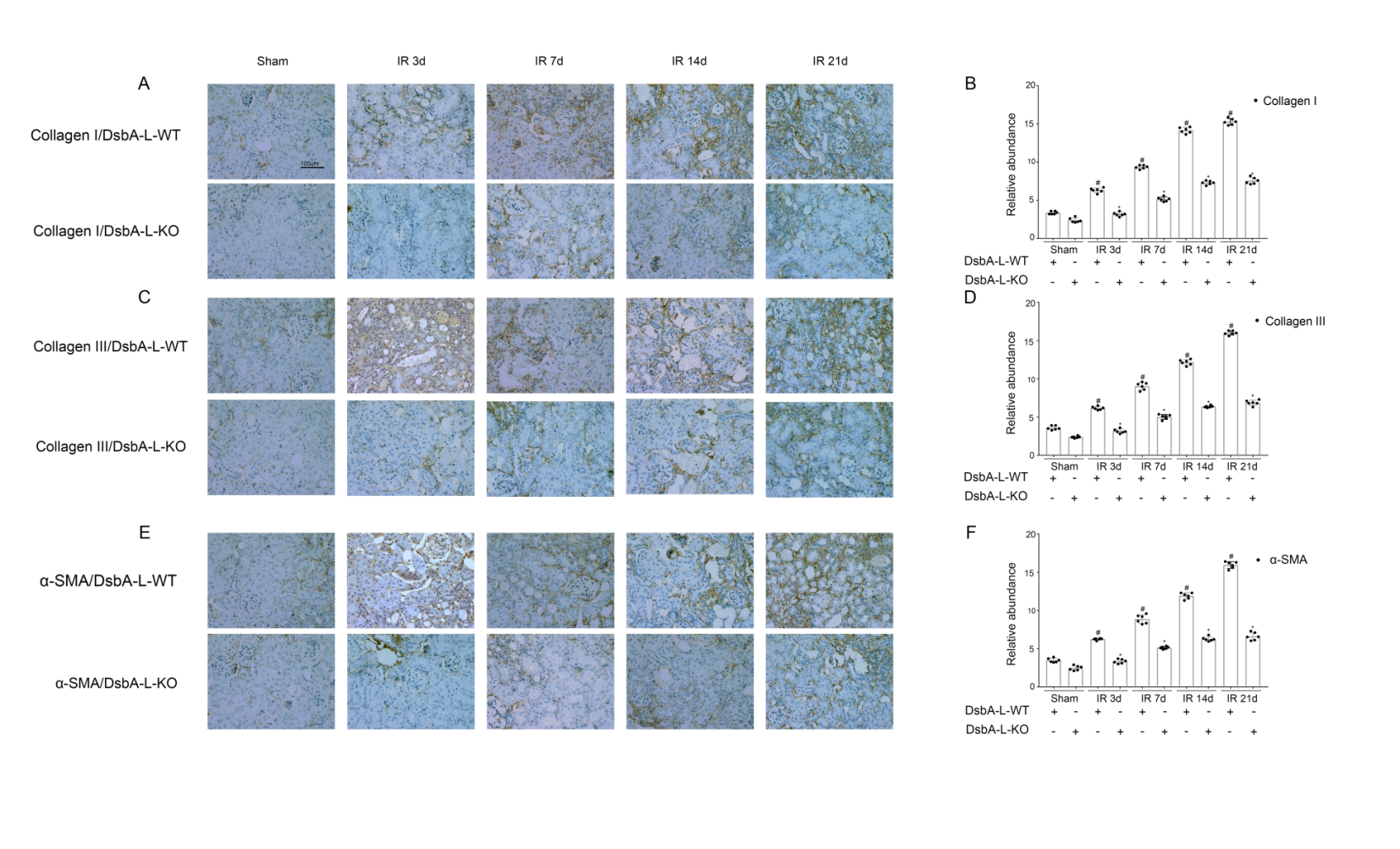


**Supplementary Figure 12:PT-DsbA-L-KO attenuated the I/R-induced** **the expression of Col 1&III, and ɑ-SMA in mice**. The PT-DsbA-L-KO and PT-DsbA-L-WT littermate mice were subjected to 28 minutes of bilateral renal ischemia followed by 3–21 days of reperfusion. (A) Immunohistochemistry analysis of Col 1&III, and ɑ-SMA. (B-F) Quantification of immunohistochemical staining. These data are representative of at least four separate experiments shown as means±sd (n=6). # *P<0.05* versus sham group. * *P<0.05* versus I/R group. (A,C&E)Each experiment was repeated 6 times independently with similar results. (B,D&F)indicate the statistical Student's T test used(means ± sd,n=6,P<0.05) .Original magnification, x400. Scar bar:100um


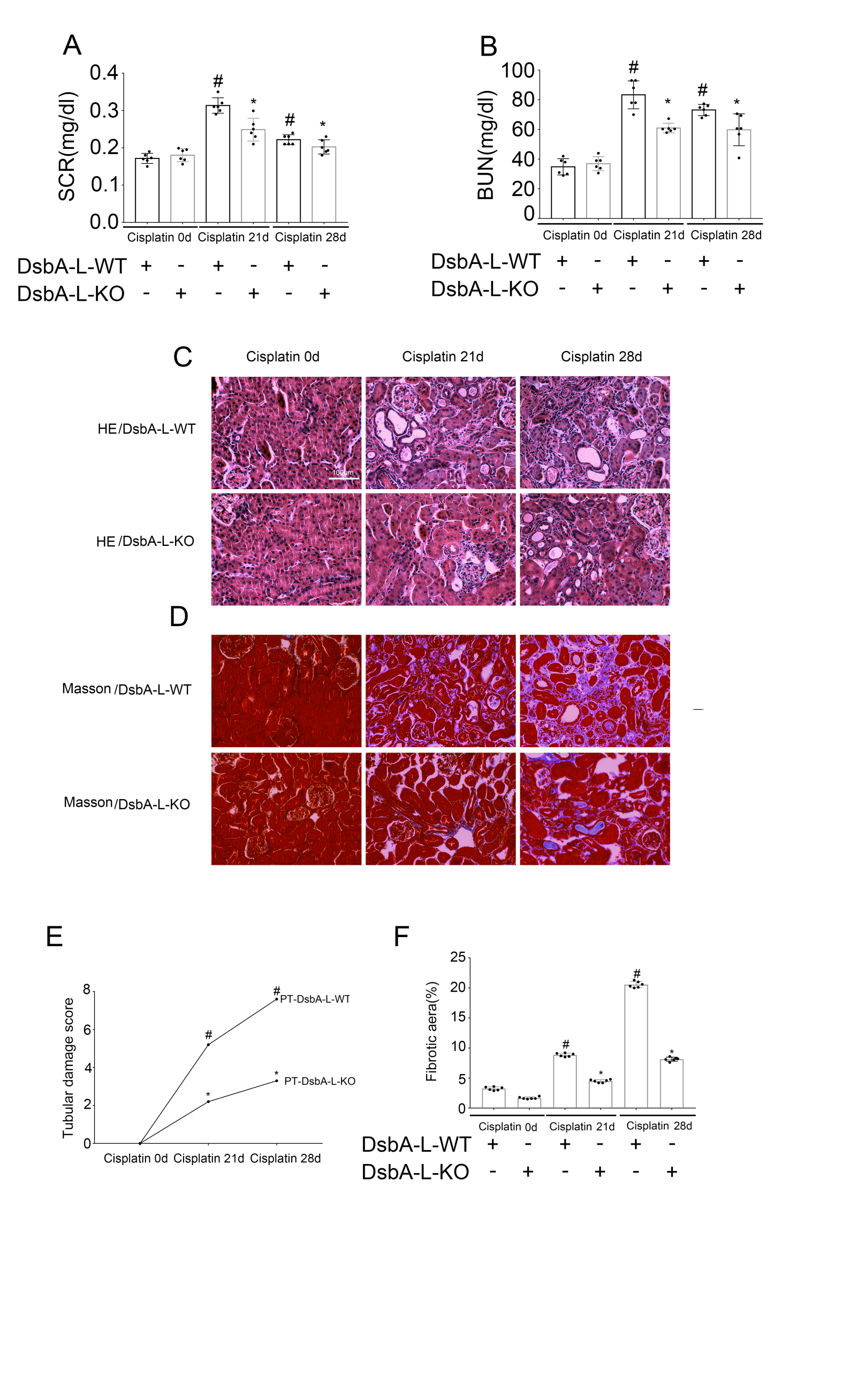


**Supplementary Figure 13**:**PT-DsbA-L-KO attenuated the low dose cisplatin-induced renal fibrosis in mice.** The PT-DsbA-L-KO and PT-DsbA-L-WT littermate mice were intraperitoneally injected with 10mg/kg cisplatin at weeks 0, 1, and 3. The blood samples were collected to measure the BUN(A) and Serum creatinine (B) at days 21 and 28. (C) Representative Hematoxylin and eosin staining. (D) Representative the Masson trichrome staining. (E) Representative the tubular damage scores. (F) Quantification of the tubulointerstitial fibrosis in the kidney cortex. These data are representative of at least four separate experiments shown as means±sd (n=6). # *P<0.05* versus Saline group. * *P<0.05* versus cisplatin group. (C-D)Each experiment was repeated 6 times independently with similar results. (A-B&E-F)indicate the statistical Student's T test used(means ± sd,n=6,P<0.05) and Original magnification, x400.


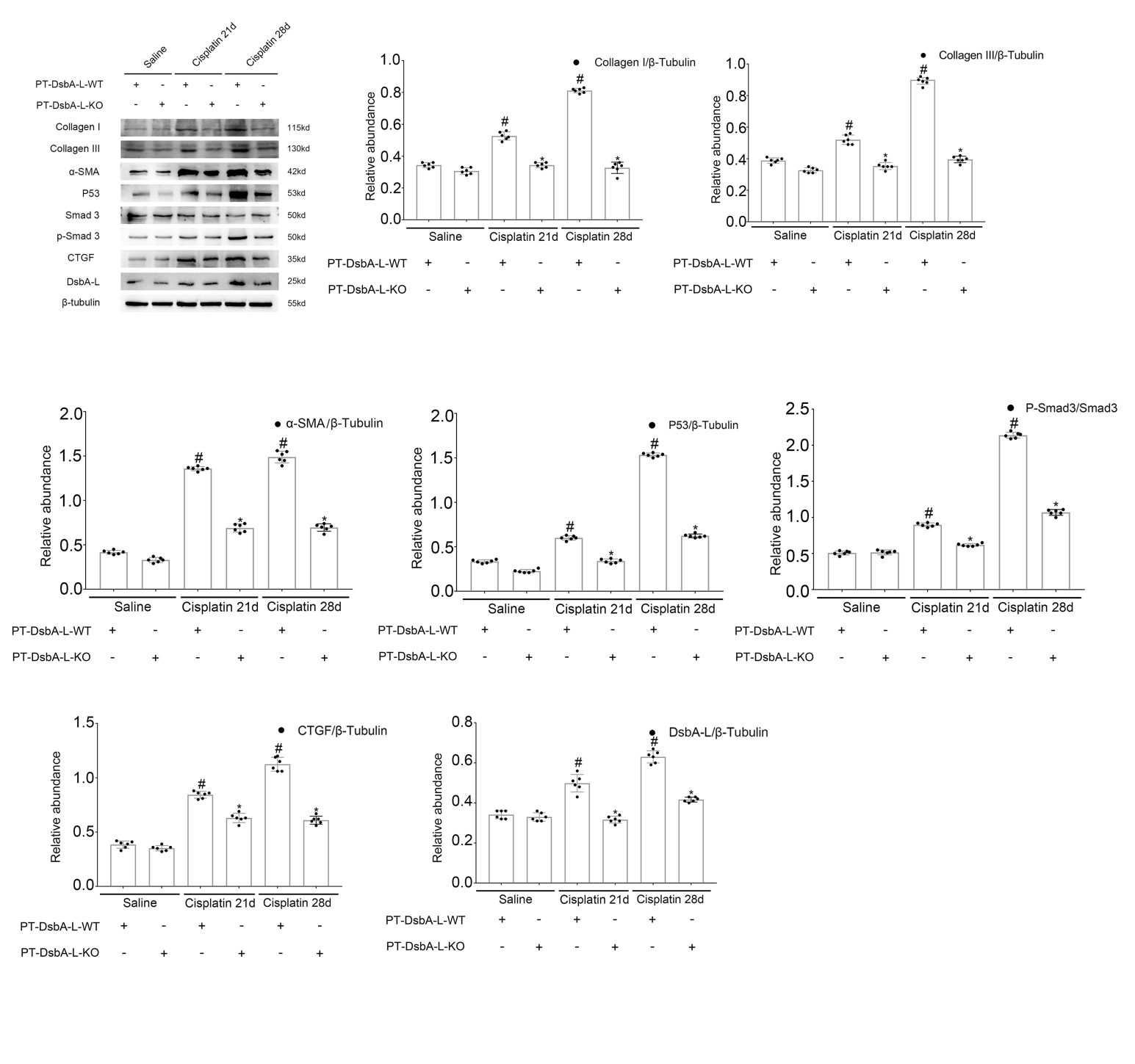


**Supplementary Figure 14**:**PT-DsbA-L-KO attenuated the low dose cisplatin-induced DsbA-L/HSP90/p53 and Smad3/CTGF/ ECM axis in mice.** The PT-DsbA-L-KO and PT-DsbA-L-WT littermate mice were intraperitoneally injected with 10mg/kg cisplatin at weeks 0, 1, and 3. (A) Immunoblot analysis of Col 1&III, ɑ-SMA, CTGF, p53, Smad3, p-Smad3, CTGF, HSP90, and β-tubulin. (A)Each experiment was repeated 6 times independently with similar results. (B-H) Analysis of the grayscale image between them. These data are representative of at least four separate experiments shown as means±sd (n=6). # *P<0.05* versus Saline group. * *P<0.05* versus cisplatin group. (B-H) indicate the statistical Student's T test used(means ± sd,n=6,P<0.05) .


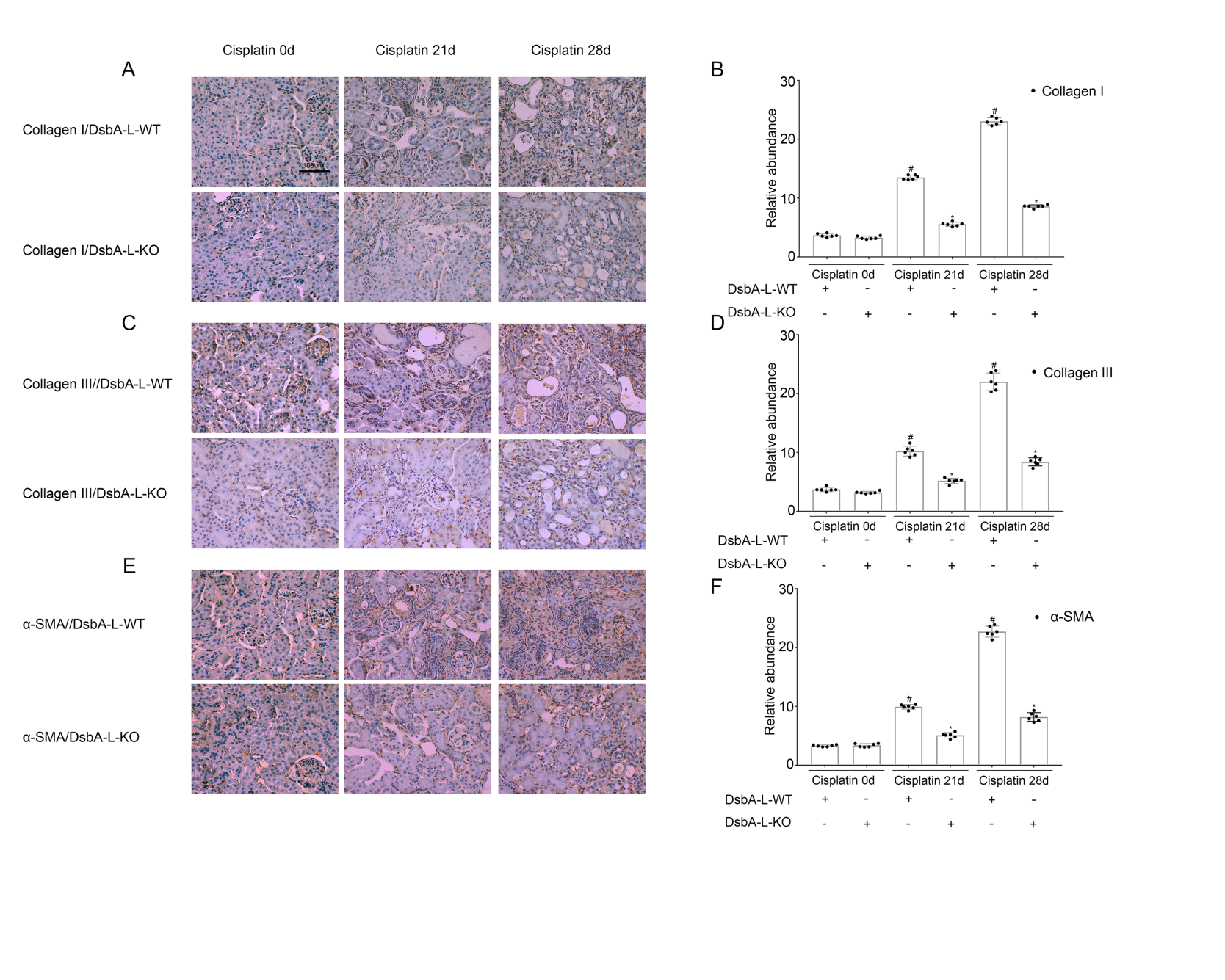


**Supplementary Figure 15**:**PT-DsbA-L-KO attenuated the low dose cisplatin-induced the expression of Col 1&III, and ɑ-SMA in mice.** The PT-DsbA-L-KO and PT-DsbA-L-WT littermate mice were intraperitoneally injected with 10mg/kg cisplatin at weeks 0, 1, and 3. (A,C&E)Immunohistochemistry analysis of Col 1&III, and ɑ-SMA. （A,C&E）Each experiment was repeated 6 times independently with similar results. (B,D&F) Quantification of immunohistochemical staining. These data are representative of at least four separate experiments shown as means±sd (n=6). # *P<0.05* versus Saline group. * *P<0.05* versus cisplatin group. (B,D&F)indicate the statistical Student's T test used(means ± sd,n=6,P<0.05) .Original magnification, x400. Scar Bar:100um


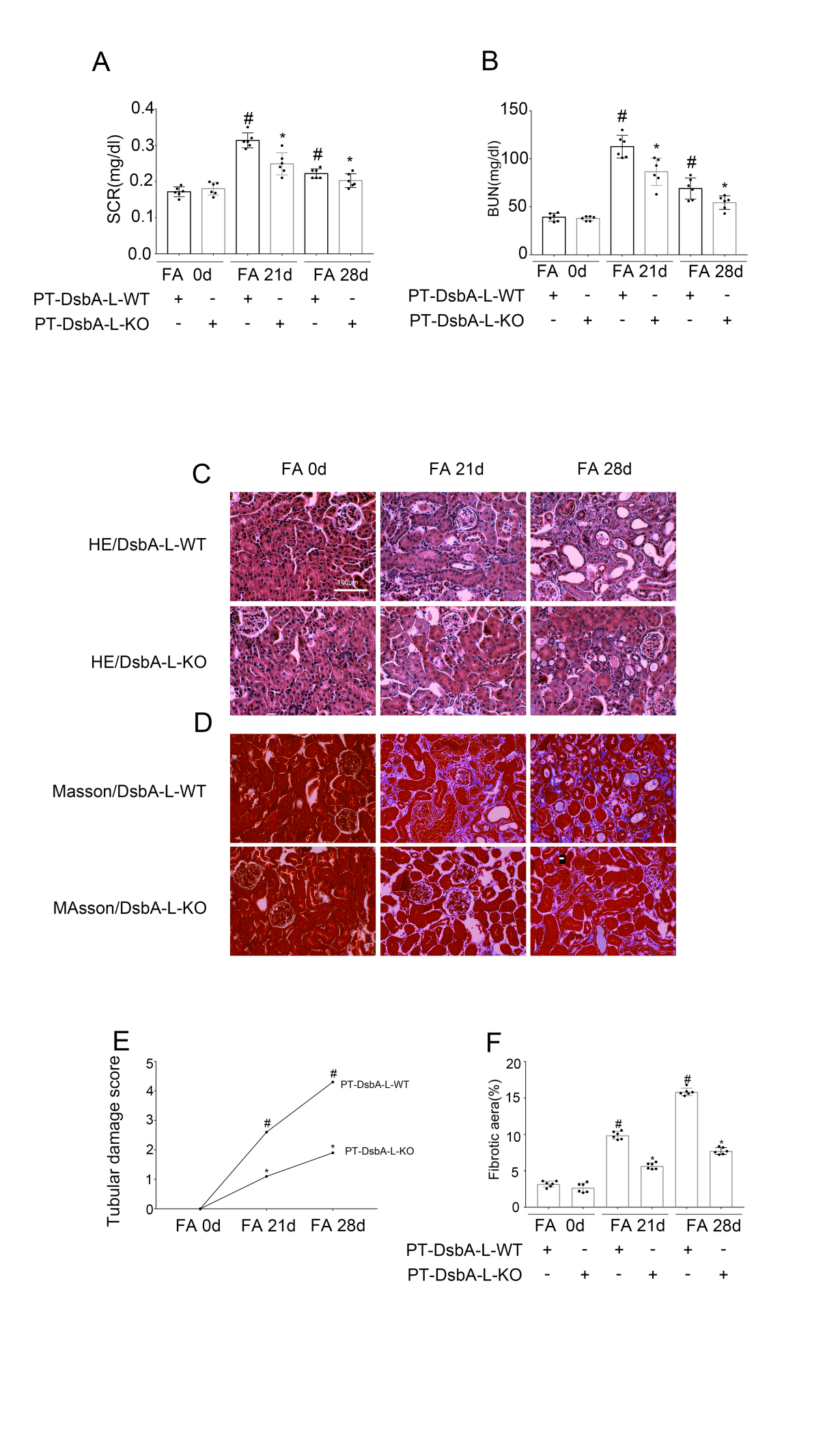


**Supplementary Figure 16**:**PT-DsbA-L-KO attenuated the low dose cisplatin-induced renal fibrosis in mice.** The PT-DsbA-L-KO and PT-DsbA-L-WT littermate mice were signal intraperitoneal injected with 250mg/kg aristolochic acid. The blood samples were collected to measure the BUN(A) and Serum creatinine (B) at days 21 and 28. (C) Representative Hematoxylin and eosin staining. (D) Representative the Masson trichrome staining. (E) Representative the tubular damage scores. (F) Quantification of the tubulointerstitial fibrosis in the kidney cortex. These data are representative of at least four separate experiments shown as means±sd (n=6). # *P<0.05* versus Saline group. * *P<0.05* versus aristolochic acid group. Each experiment(C,D) was repeated 6 times independently with similar results. (A-B,E-F) indicate the statistical Student's T test used(means ± sd,n=6,P<0.05). Original magnification, x400. Scar bar:100um


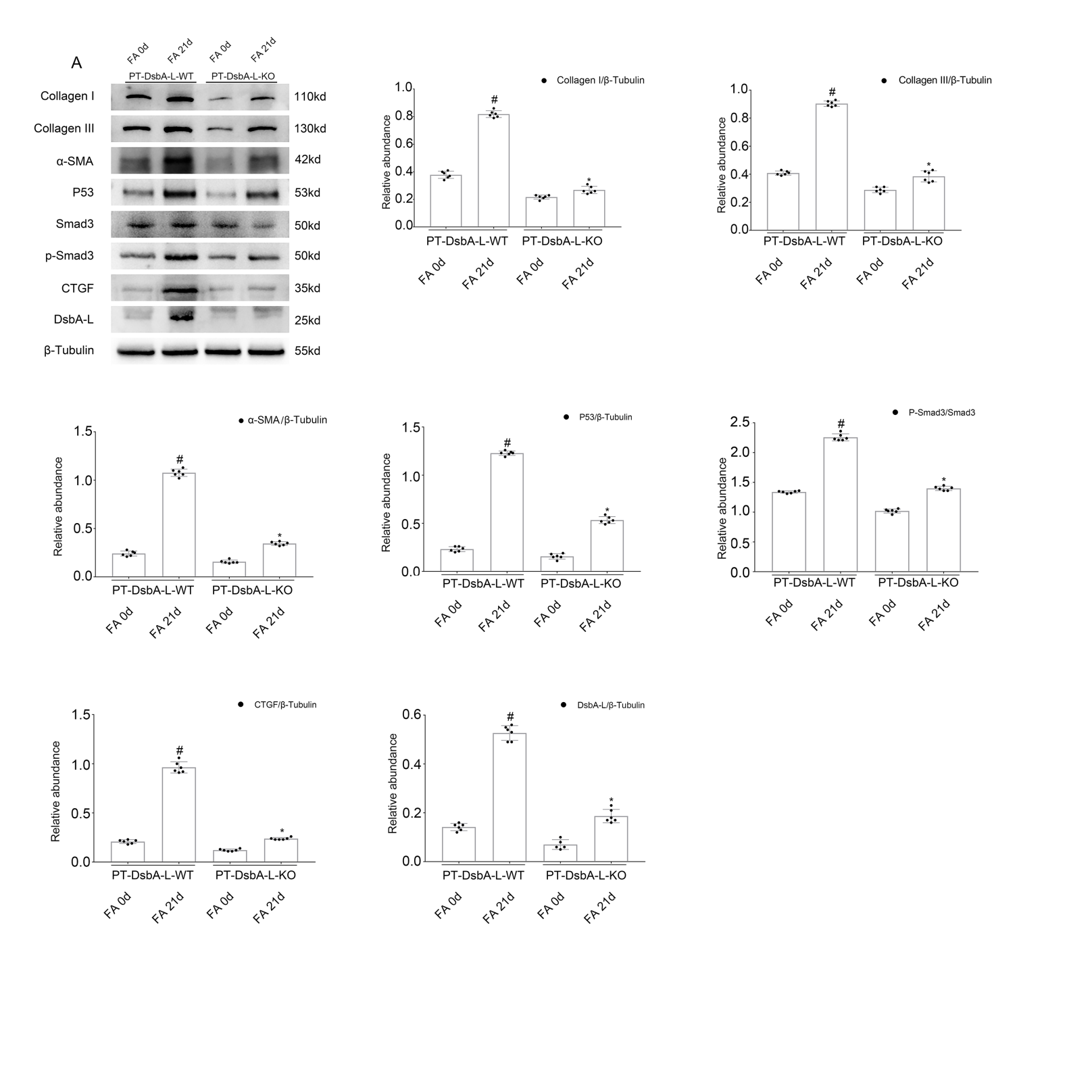


**Supplementary Figure 17**:**PT-DsbA-L-KO attenuated the aristolochic acid-induced DsbA-L/HSP90/p53 and Smad3/CTGF/ ECM axis in mice at days 21.** The PT-DsbA-L-KO and PT-DsbA-L-WT littermate mice were signal intraperitoneal injected with 250mg/kg aristolochic acid. (A) Immunoblot analysis of Col 1&III, ɑ-SMA, CTGF, p53, Smad3, p-Smad3, CTGF, HSP90, and β-tubulin at days 21. (B-H) Analysis of the grayscale image between them. These data are representative of at least four separate experiments shown as means±sd (n=6). # *P<0.05* versus Saline group. * *P<0.05* versusaristolochic acid group. Each experiment(A) was repeated 6 times independently with similar results. (B-H) indicate the statistical Student's T test used(means ± sd,n=6,P<0.05).


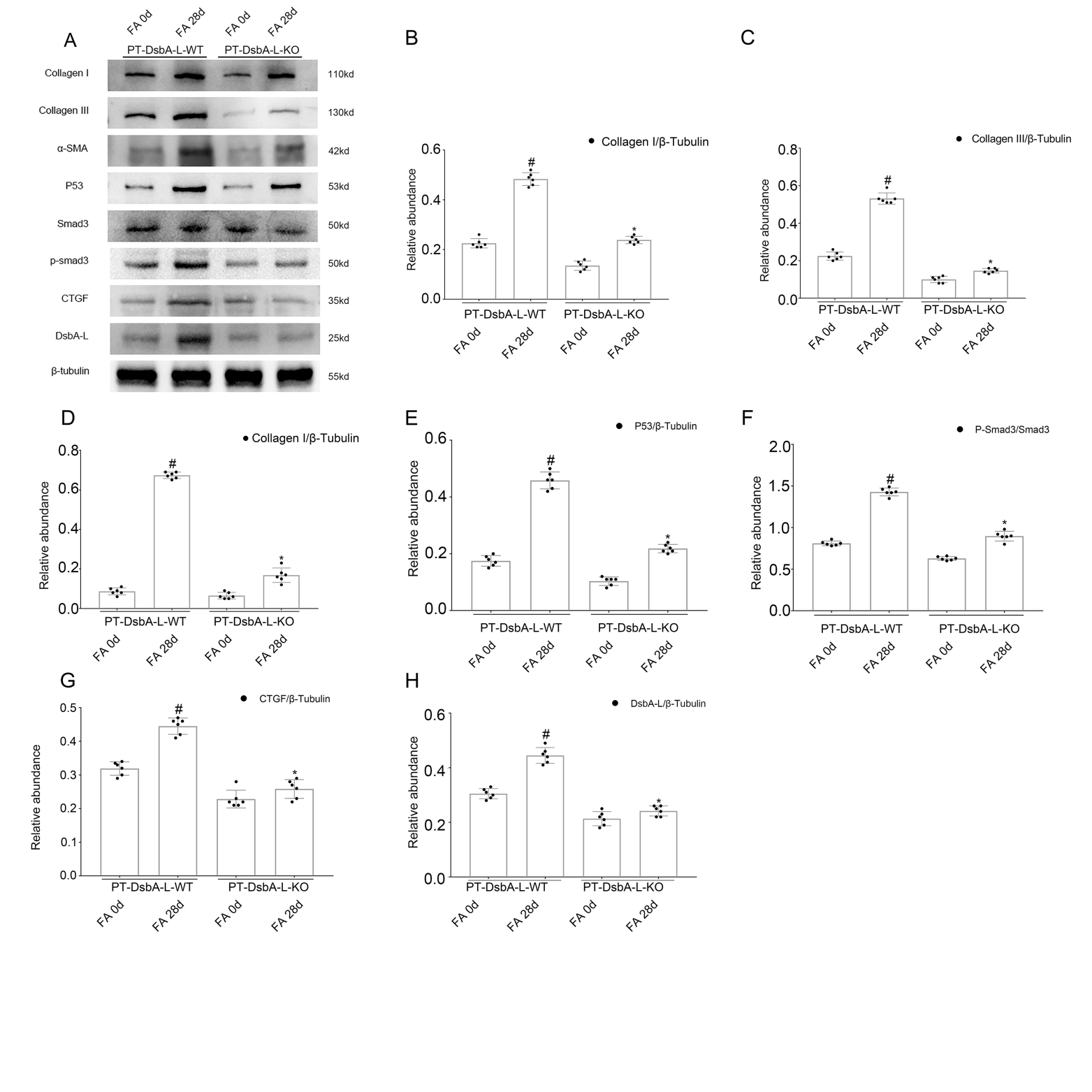


**Supplementary Figure 18**:**PT-DsbA-L-KO attenuated the aristolochic acid-induced DsbA-L/HSP90/p53 and Smad3/CTGF/ ECM axis in mice at days 28.** The PT-DsbA-L-KO and PT-DsbA-L-WT littermate mice were signal intraperitoneal injected with 250mg/kg aristolochic acid. (A) Immunoblot analysis of Col 1&III, ɑ-SMA, CTGF, p53, Smad3, p-Smad3, CTGF, HSP90, and β-tubulin at days 28. (B-H) Analysis of the grayscale image between them. These data are representative of at least four separate experiments shown as means±sd (n=6). # *P<0.05* versus Saline group. * *P<0.05* versus aristolochic acid group. Each experiment(A) was repeated 6 times independently with similar results. (B-H) indicate the statistical Student's T test used(means ± sd,n=6,P<0.05).


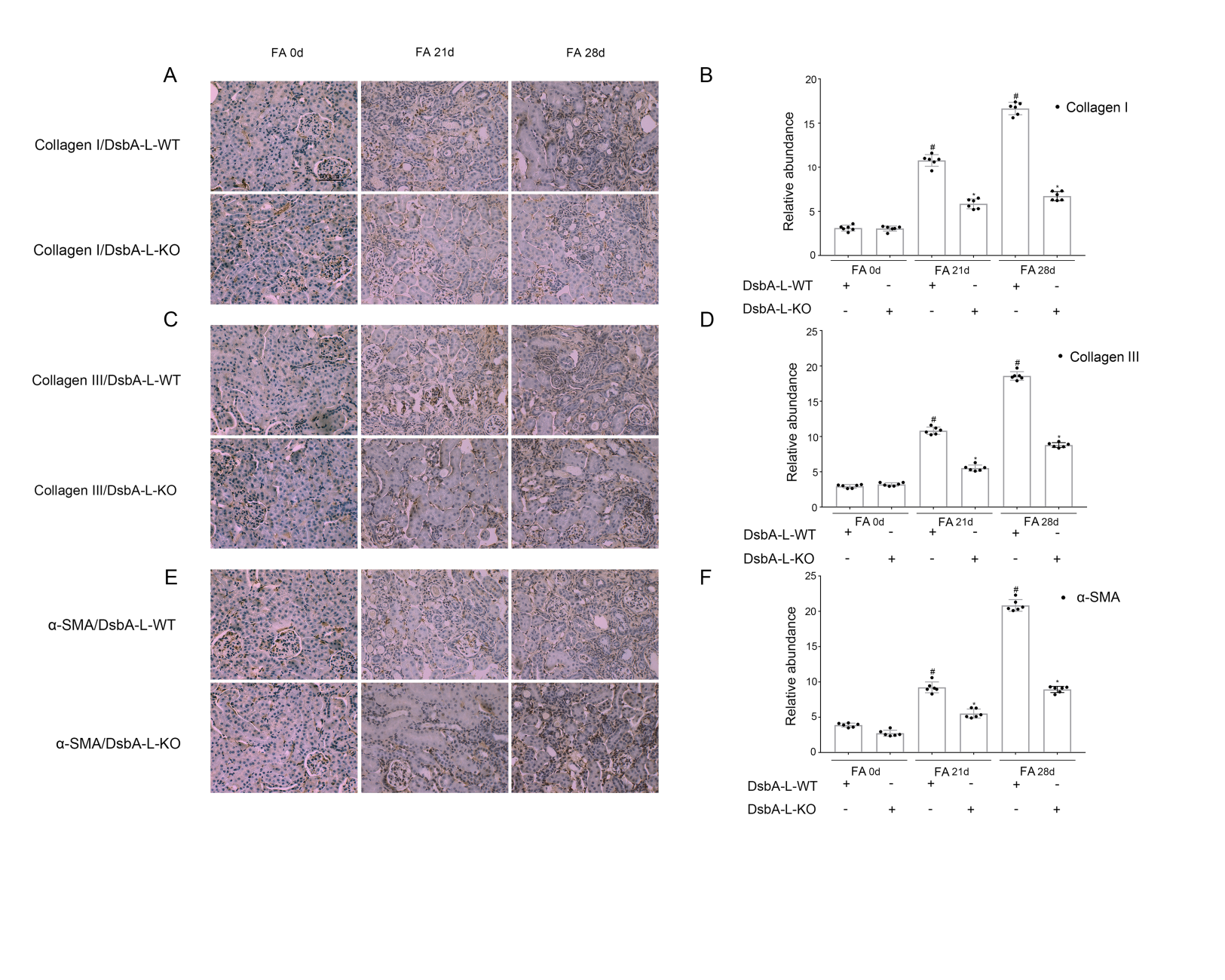


**Supplementary Figure 19**:**PT-DsbA-L-KO attenuated the aristolochic acid-induced the expression of Col 1&III, and ɑ-SMA in mice.** The PT-DsbA-L-KO and PT-DsbA-L-WT littermate mice were signal intraperitoneal injected with 250mg/kg aristolochic acid. (A,C, and E) Immunohistochemistry analysis of Col 1&III, and ɑ-SMA at days 21 and 28. (B, D, and F) Quantification of immunohistochemical staining. These data are representative of at least four separate experiments shown as means±sd (n=6). # *P<0.05* versus Saline group. * *P<0.05* versus aristolochic acid group. Each experiment(A,C&E) was repeated 6 times independently with similar results. (B,D&F) indicate the statistical Student's T test used(means ± sd,n=6,P<0.05). Original magnification, x400. Scar bar:100um


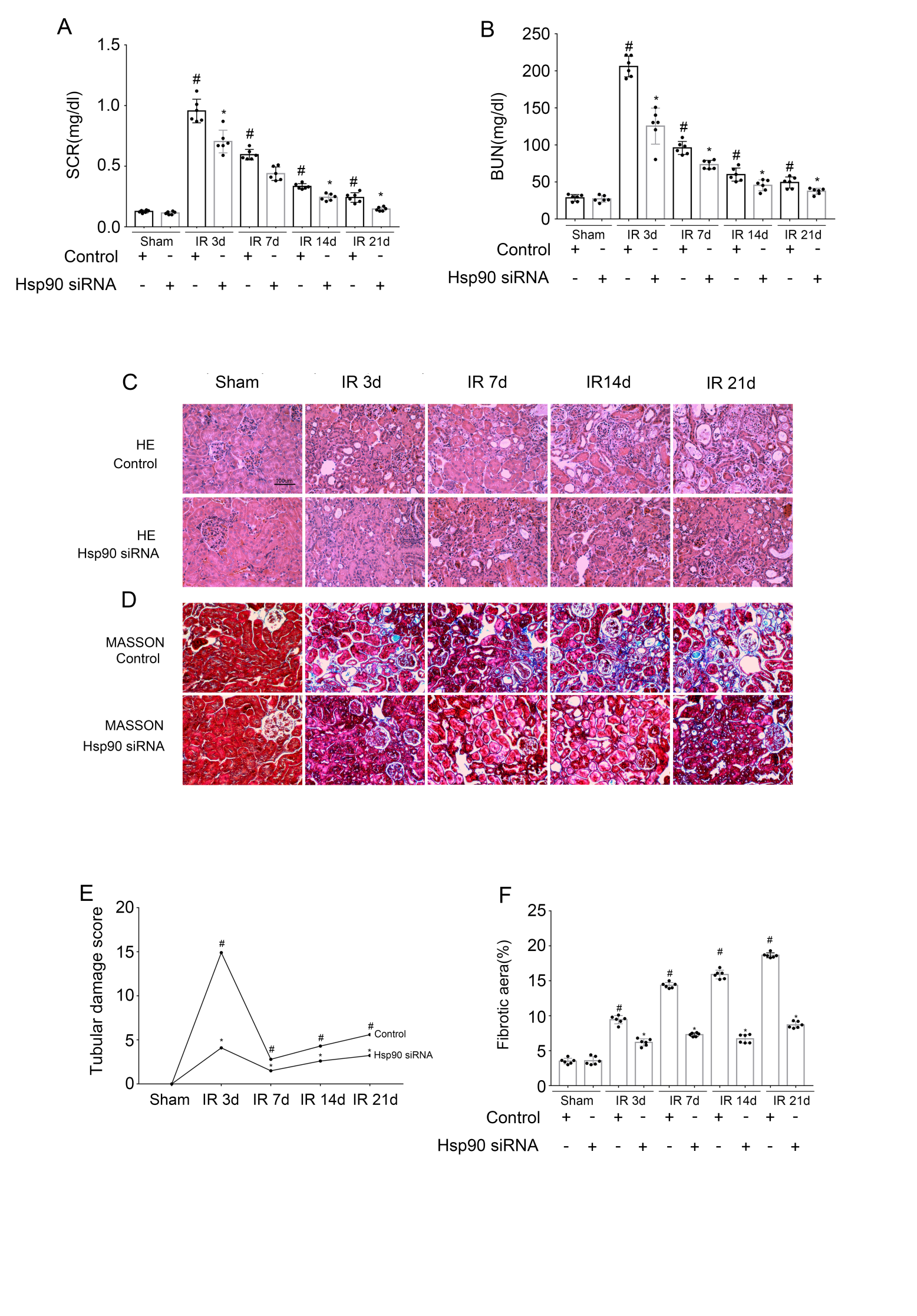


**Supplementary Figure 20**:Hsp90β siRNA **attenuated the I/R-induced renal fibrosis in mice.** Male C57BL/6 mice were subjected to 28 minutes of bilateral renal ischemia and treatment with or without 15mg/kg Hsp90β siRNA twice a week, and followed by 3–21 days of reperfusion. The blood samples were collected to measure the BUN(A) and Serum creatinine (B). (C) Representative Hematoxylin and eosin staining. (D) Representative the Masson trichrome staining. (E) Representative the tubular damage scores. (F) Quantification of the tubulointerstitial fibrosis in the kidney cortex. These data are representative of at least four separate experiments shown as means±sd (n=6). # *P<0.05* versus sham group. * *P<0.05* versus I/R group. Each experiment(C,D) was repeated 6 times independently with similar results. (A-B,E-F) indicate the statistical Student's T test used(means ± sd,n=6,P<0.05). Original magnification, x400. Scar Bar:100um


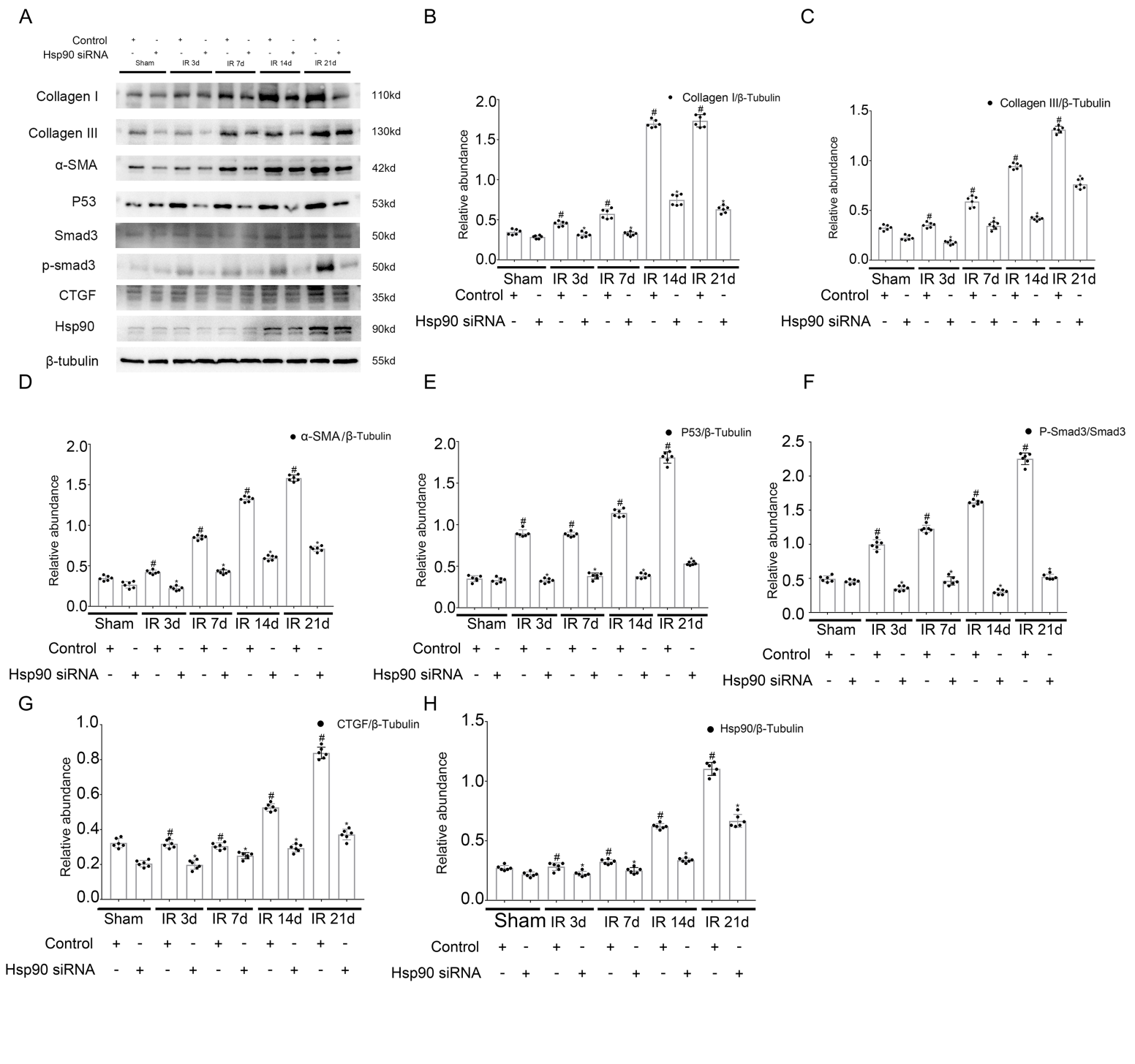


**Supplementary Figure 21**:**Hsp90β siRNA attenuated the I/R-induced HSP90/p53 and Smad3/CTGF/ ECM axis in mice.** Male C57BL/6 mice were subjected to 28 minutes of bilateral renal ischemia and treatment with or without 15mg/kg Hsp90β siRNA twice a week, and followed by 3–21 days of reperfusion. (A) Immunoblot analysis of Col 1&III, ɑ-SMA, CTGF, p53, Smad3, p-Smad3, CTGF, HSP90, and β-tubulin. (B-H) Analysis of the grayscale image between them. These data are representative of at least four separate experiments shown as means±sd (n=6). # *P<0.05* versus sham group. * *P<0.05* versus I/R group. Each experiment(A) was repeated 6 times independently with similar results. (B-H) indicate the statistical Student's T test used(means ± sd,n=6,P<0.05).


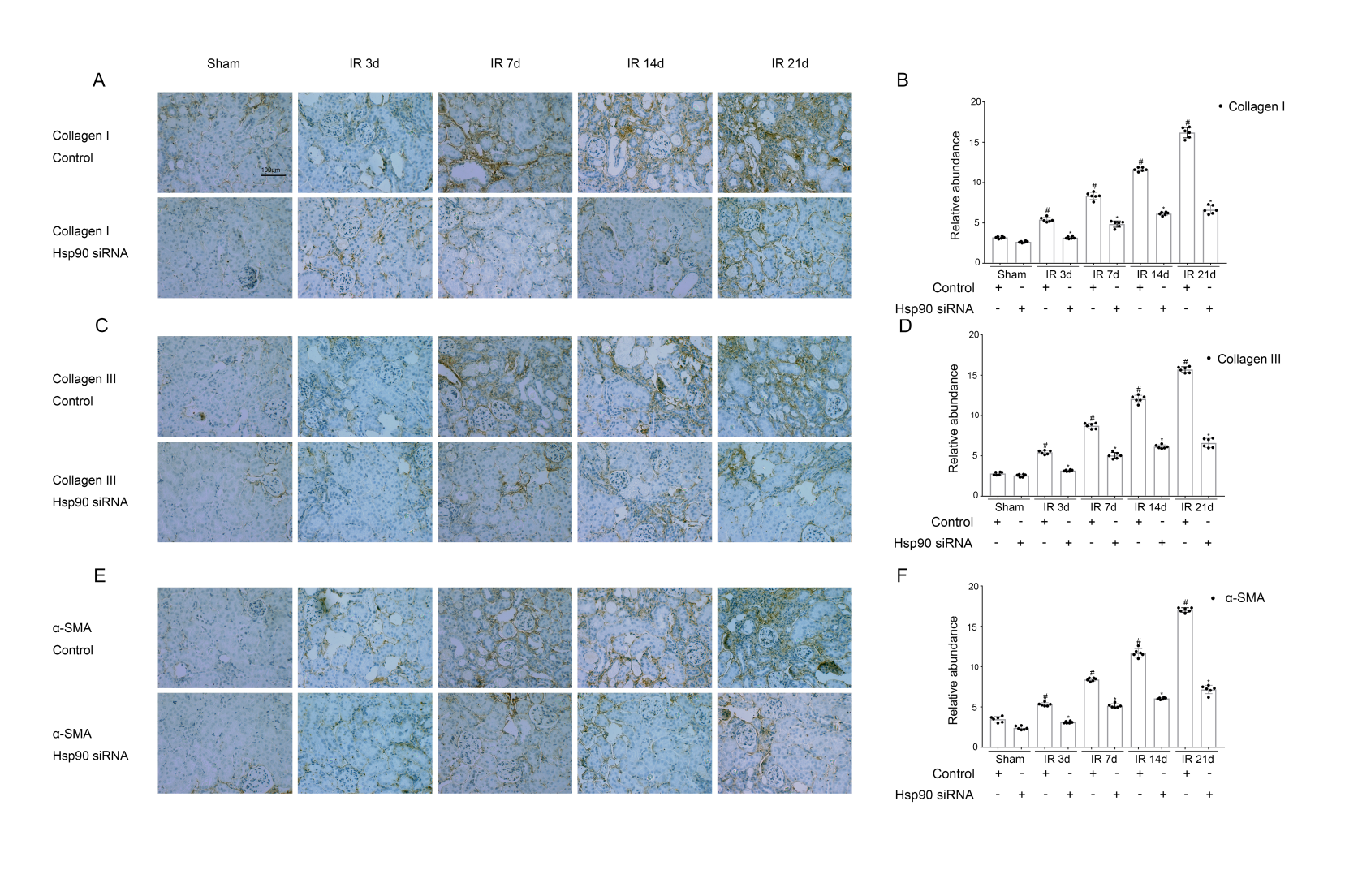


**Supplementary Figure 22:Hsp90β siRNA attenuated the I/R-induced the expression of Col 1&III, and ɑ-SMA in mice**.Male C57BL/6 mice were subjected to 28 minutes of bilateral renal ischemia and treatment with or without 15mg/kg Hsp90β siRNA twice a week, and followed by 3–21 days of reperfusion. (A) Immunohistochemistry analysis of Col 1&III, and ɑ-SMA. (B-F) Quantification of immunohistochemical staining. These data are representative of at least four separate experiments shown as means±sd (n=6). # *P<0.05* versus sham group. * *P<0.05* versus I/R group. Each experiment(A,C&E) was repeated 6 times independently with similar results. (B,D&F) indicate the statistical Student's T test used(means ± sd,n=6,P<0.05). Original magnification, x400. Scar bar:100um


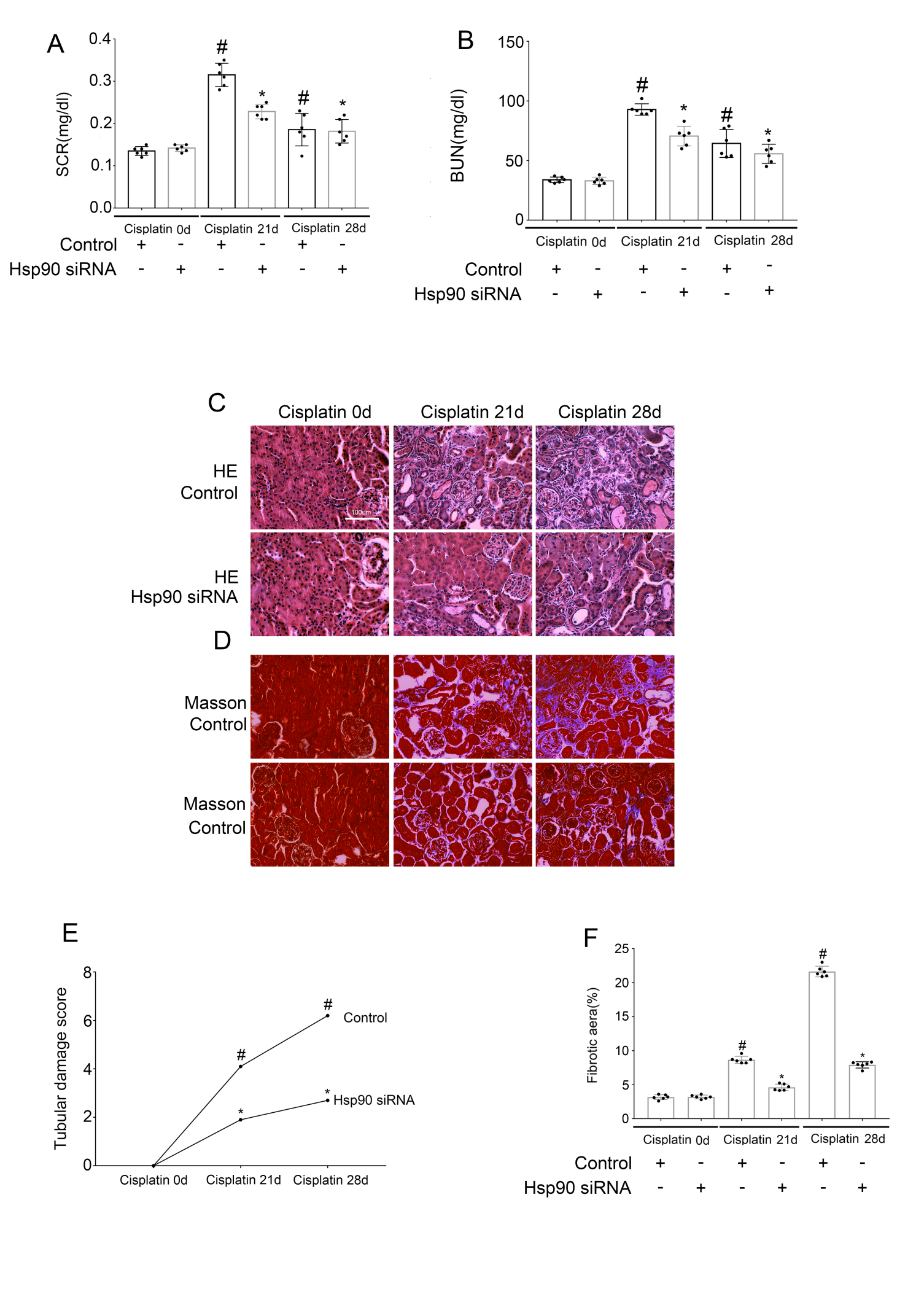


**Supplementary Figure 23**:**Hsp90β siRNA attenuated the low dose cisplatin-induced renal fibrosis in mice.** Male C57BL/6 mice were intraperitoneally injected with 10mg/kg cisplatin at weeks 0, 1, and 3, and then plus with or without 15mg/kg Hsp90β siRNA twice a week.The blood samples were collected to measure the BUN(A) and Serum creatinine (B) at days 21 and 28. (C) Representative Hematoxylin and eosinstaining. (D) Representative the Masson trichrome staining. (E) Representative the tubular damage scores. (F) Quantification of the tubulointerstitial fibrosis in the kidney cortex. These data are representative of at least four separate experiments shown as means±sd (n=6). # *P<0.05* versus Saline group. * *P<0.05* versus cisplatin group. Each experiment(C,D) was repeated 6 times independently with similar results. (A-B,E-F) indicate the statistical Student's T test used(means ± sd,n=6,P<0.05). Original magnification, x400. Scar bar:100um


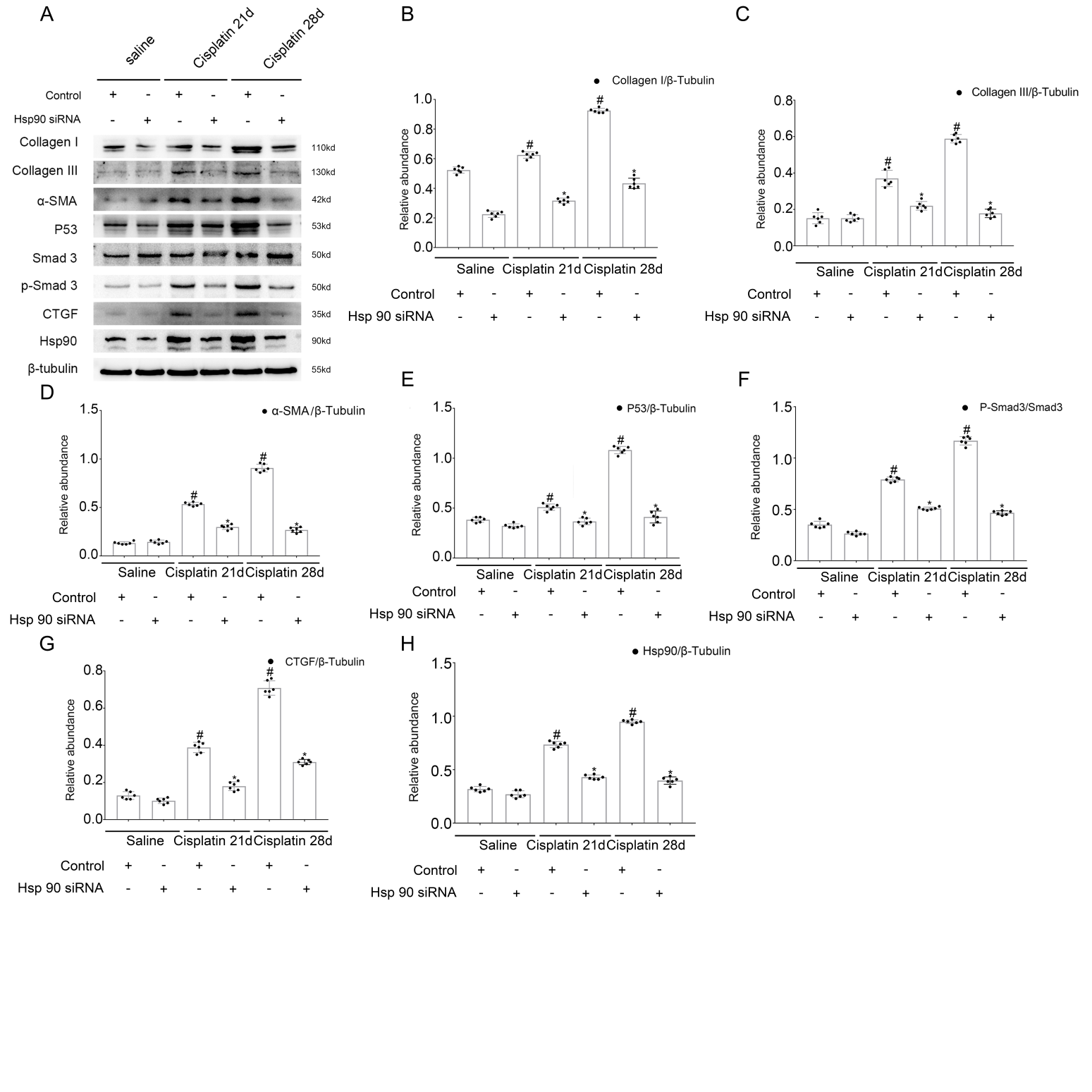


**Supplementary Figure 24**:**Hsp90β siRNA attenuated** **the low dose cisplatin-induced HSP90/p53 and Smad3/CTGF/ ECM axis in mice.** Male C57BL/6 mice were intraperitoneally injected with 10mg/kg cisplatin at weeks 0, 1, and 3, and then plus with or without 15mg/kg Hsp90β siRNA twice a week. (A) Immunoblot analysis of Col 1&III, ɑ-SMA, CTGF, p53, Smad3, p-Smad3, CTGF, HSP90, and β-tubulin at days 21 and 28. (B-H) Analysis of the grayscale image between them. These data are representative of at least four separate experiments shown as means±sd (n=6). # *P<0.05* versus Saline group. * *P<0.05* versus cisplatin group. Each experiment(A) was repeated 6 times independently with similar results. (B-H) indicate the statistical Student's T test used(means ± sd,n=6,P<0.05).


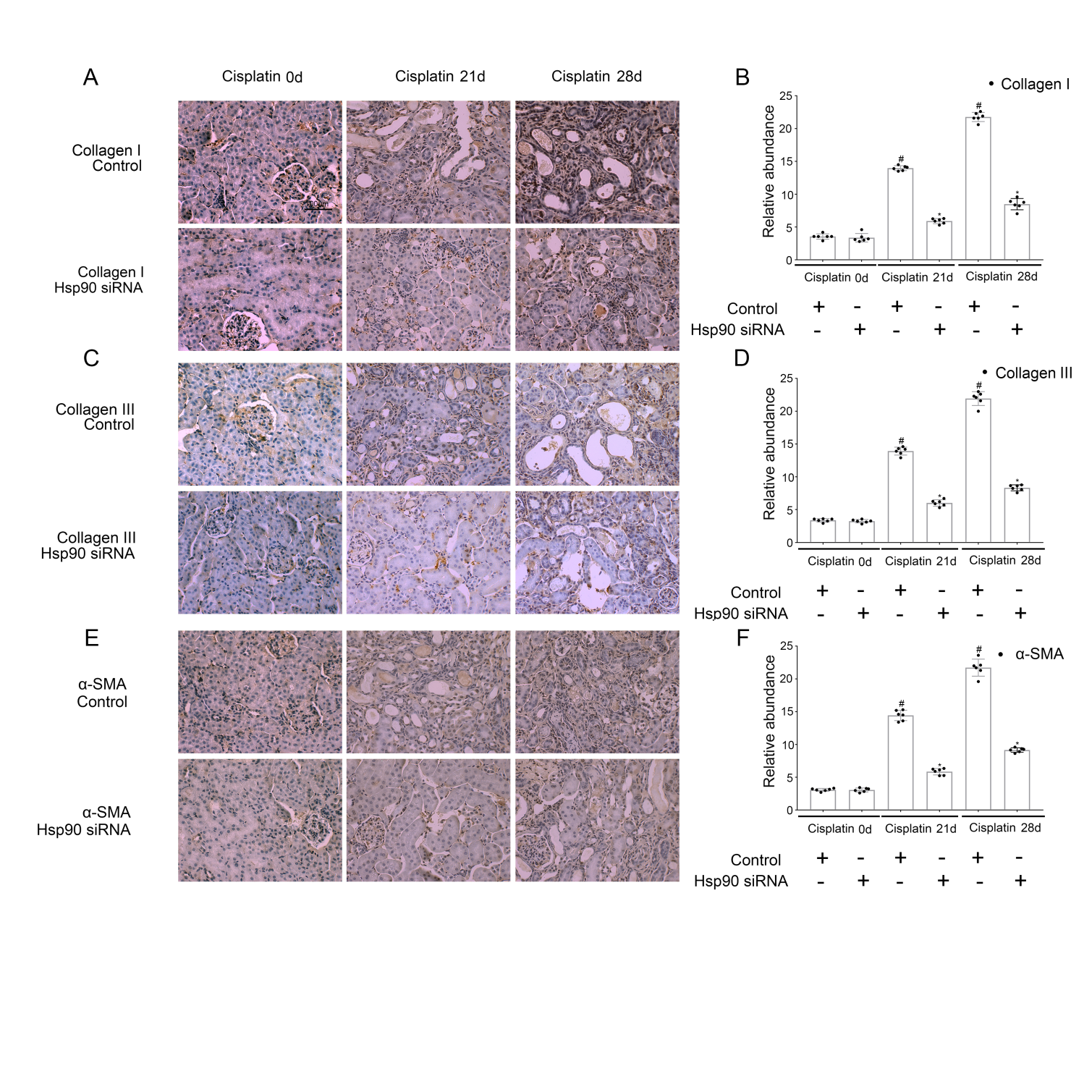


**Supplementary Figure 25: Hsp90β siRNA attenuated the low dose cisplatin-****induced the expression of Col 1&III, and ɑ-SMA in mice**. Male C57BL/6 mice were intraperitoneally injected with 10mg/kg cisplatin at weeks 0, 1, and 3, and then plus with or without 15mg/kg Hsp90β siRNA twice a week. (A) Immunohistochemistry analysis of Col 1&III, and ɑ-SMA at days 21 and 28. (B-F) Quantification of immunohistochemical staining. These data are representative of at least four separate experiments shown as means±sd (n=6). # *P<0.05* versus sham group. * *P<0.05* versus I/R group. Each experiment(A,C&E) was repeated 6 times independently with similar results. (B,D&F) indicate the statistical Student's T test used(means ± sd,n=6,P<0.05). Original magnification, x400. Scar bar:100um


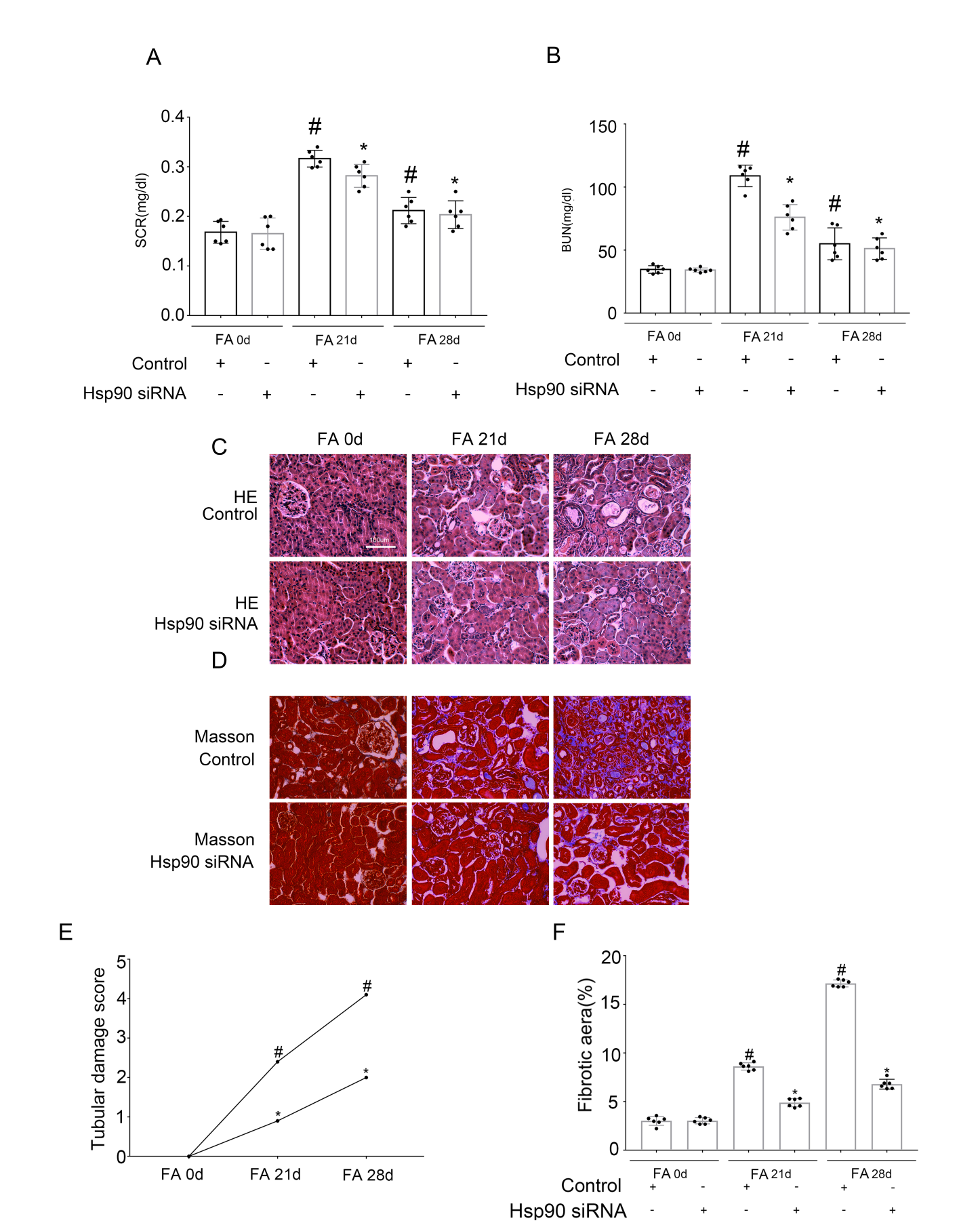


**Supplementary Figure 26: Hsp90β siRNA attenuated the low dose cisplatin-induced renal fibrosis in mice.** Male C57BL/6 mice were signal intraperitoneal injected with 250mg/kg aristolochic acid, and then plus with or without 15mg/kg Hsp90β siRNA twice a week. The blood samples were collected to measure the BUN(A) and Serum creatinine (B) at days 21 and 28. (C) Representative Hematoxylin and eosin staining. (D) Representative the Masson trichrome staining. (E) Representative the tubular damage scores. (F) Quantification of the tubulointerstitial fibrosis in the kidney cortex. These data are representative of at least four separate experiments shown as means±sd (n=6). # *P<0.05* versus Saline group. * *P<0.05* versus aristolochic acid group. Each experiment(C,D) was repeated 6 times independently with similar results. (A-B,E-F) indicate the statistical Student's T test used(means ± sd,n=6,P<0.05). Original magnification, x400. Scar bar:100um


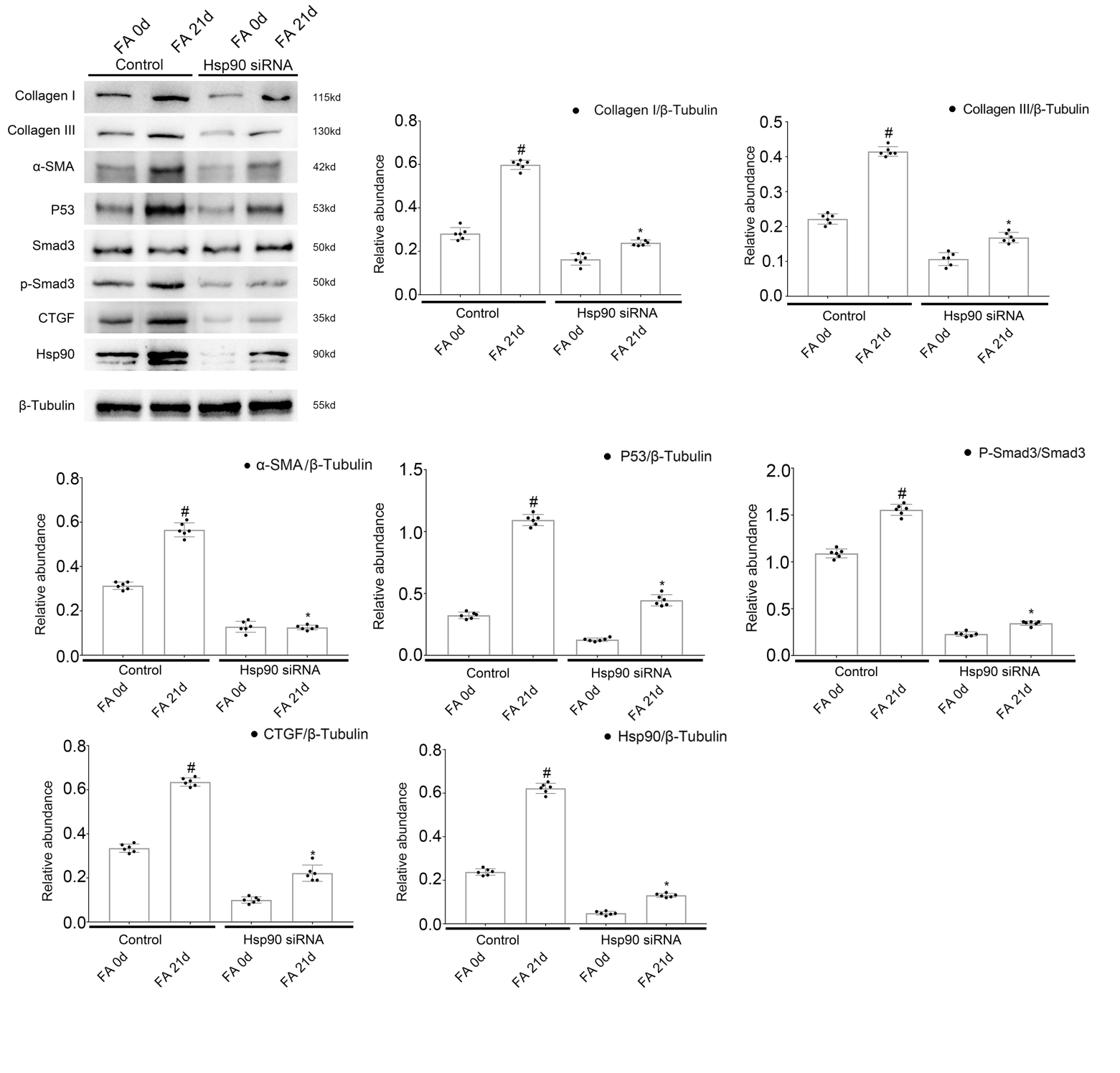


**Supplementary Figure 27**:**Hsp90β siRNA attenuated the** **aristolochic acid-induced HSP90/p53 and Smad3/CTGF/ ECM axis in mice at days 21.** Male C57BL/6 mice were signal intraperitoneal injected with 250mg/kg aristolochic acid, and then plus with or without 15mg/kg Hsp90β siRNA twice a week. (A) Immunoblot analysis of Col 1&III, ɑ-SMA, CTGF, p53, Smad3, p-Smad3, CTGF, HSP90, and β-tubulin at days 21. (B-H) Analysis of the grayscale image between them. These data are representative of at least four separate experiments shown as means±sd (n=6). # *P<0.05* versus Saline group. * *P<0.05* versus aristolochic acid group. Each experiment(A) was repeated 6 times independently with similar results. (B-H) indicate the statistical Student's T test used(means ± sd,n=6,P<0.05).


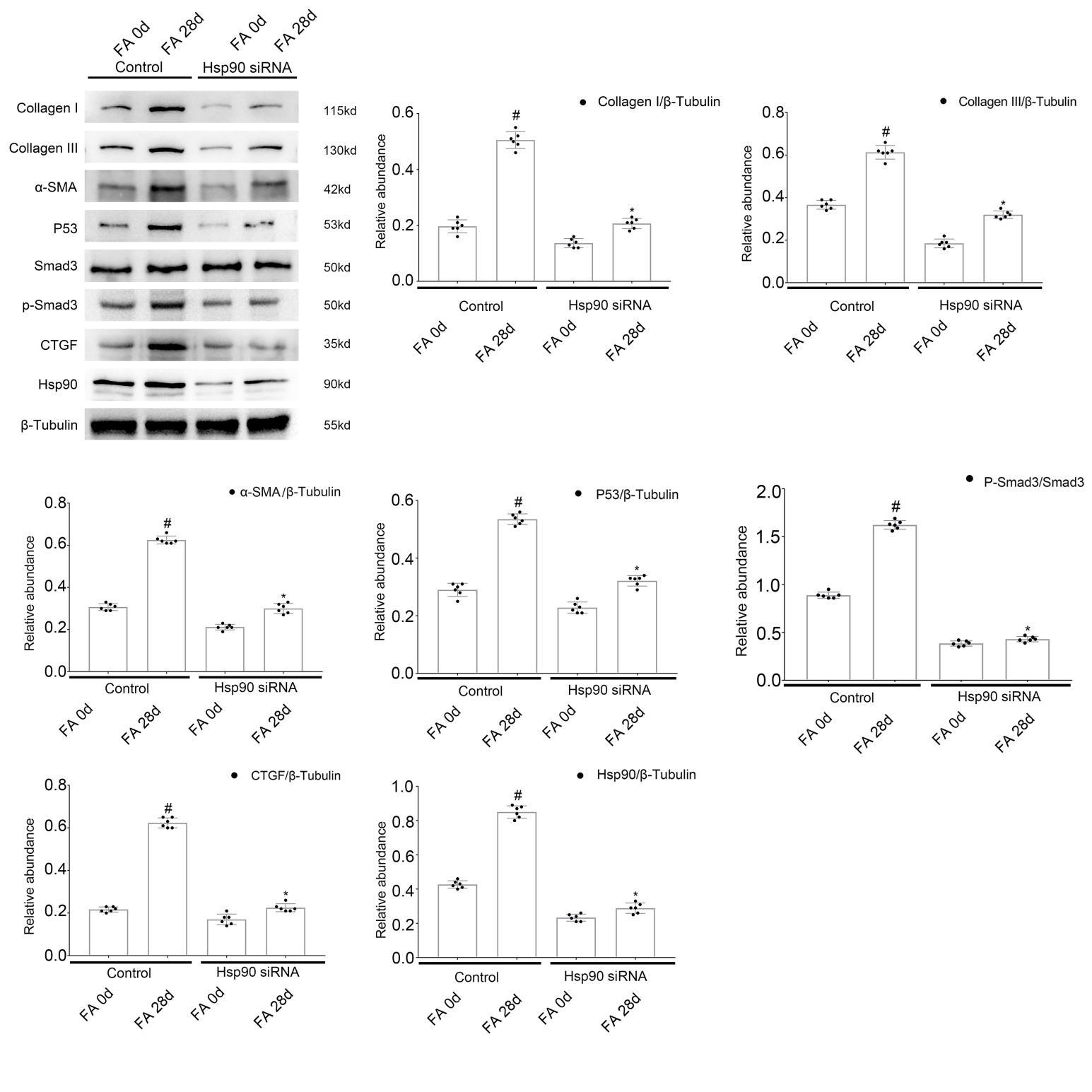


**Supplementary Figure 28**:**Hsp90βsiRNA attenuated the aristolochic acid-induced HSP90/p53 and Smad3/CTGF/ ECM axis in mice at days 28.** Male C57BL/6 mice were signal intraperitoneal injected with 250mg/kg aristolochic acid, and then plus with or without 15mg/kg Hsp90β siRNA twice a week. (A) Immunoblot analysis of Col 1&III, ɑ-SMA, CTGF, p53, Smad3, p-Smad3, CTGF, HSP90, and β-tubulin at days 28. (B-H) Analysis of the grayscale image between them. These data are representative of at least four separate experiments shown as means±sd (n=6). # *P<0.05* versus Saline group. * *P<0.05* versus aristolochic acid group. Each experiment(A) was repeated 6 times independently with similar results. (B-H) indicate the statistical Student's T test used(means ± sd,n=6,P<0.05).


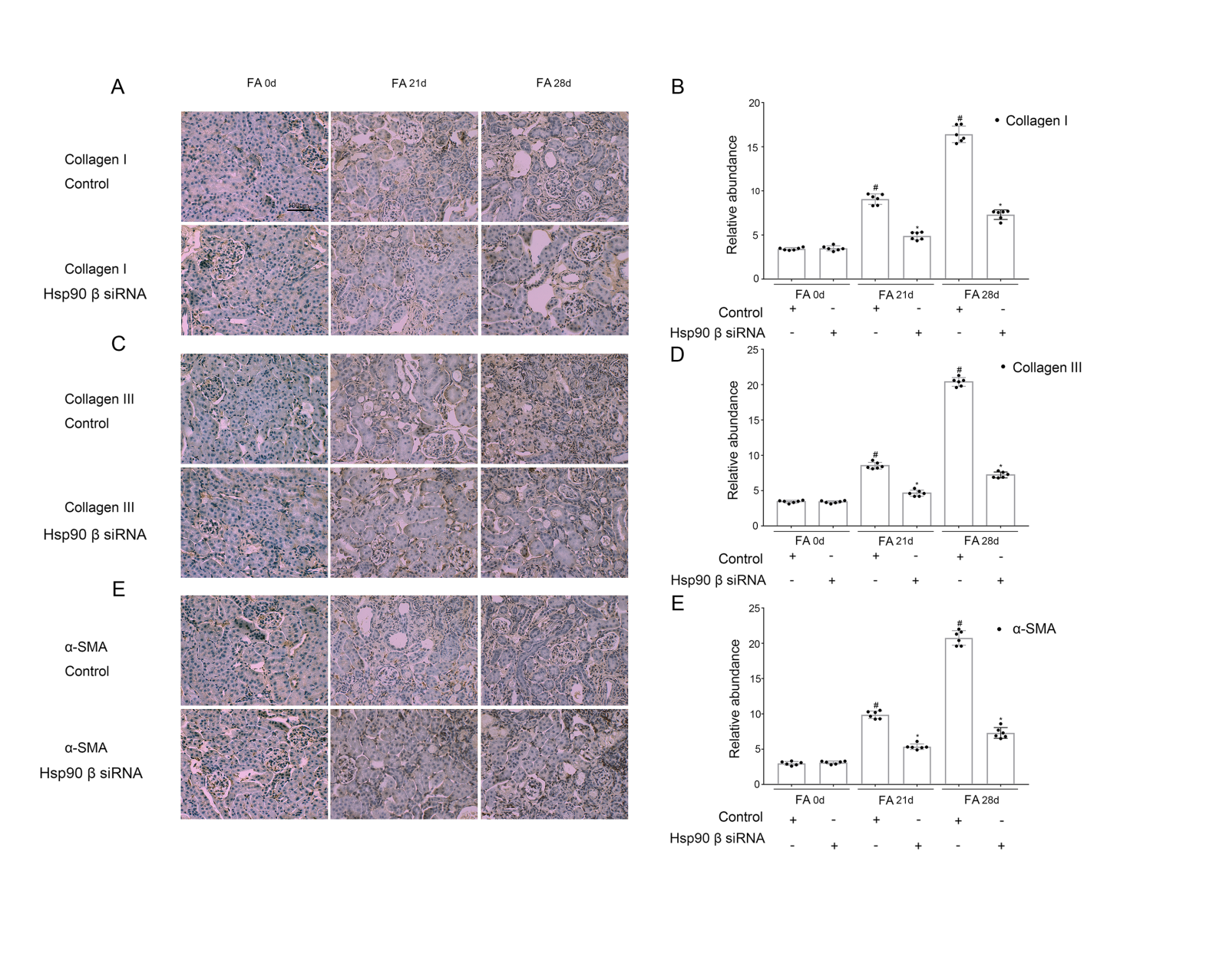


**Supplementary Figure 29**:**Hsp90β siRNA attenuated the aristolochic acid-induced the expression of Col 1&III, and ɑ-SMA in mice**. Male C57BL/6 mice were signal intraperitoneal injected with 250mg/kg aristolochic acid, and then plus with or without 15mg/kg Hsp90βsiRNA twice a week. (A) Immunohistochemistry analysis of Col 1&III, and ɑ-SMA at days 21 and 28. (B-F) Quantification of immunohistochemical staining.These data are representative of at least four separate experiments shown as means±sd (n=6). # *P<0.05* versus Saline group. * *P<0.05* versus aristolochic acid group. Each experiment(A,C&E) was repeated 6 times independently with similar results. (B,D&F) indicate the statistical Student's T test used(means ± sd,n=6,P<0.05).


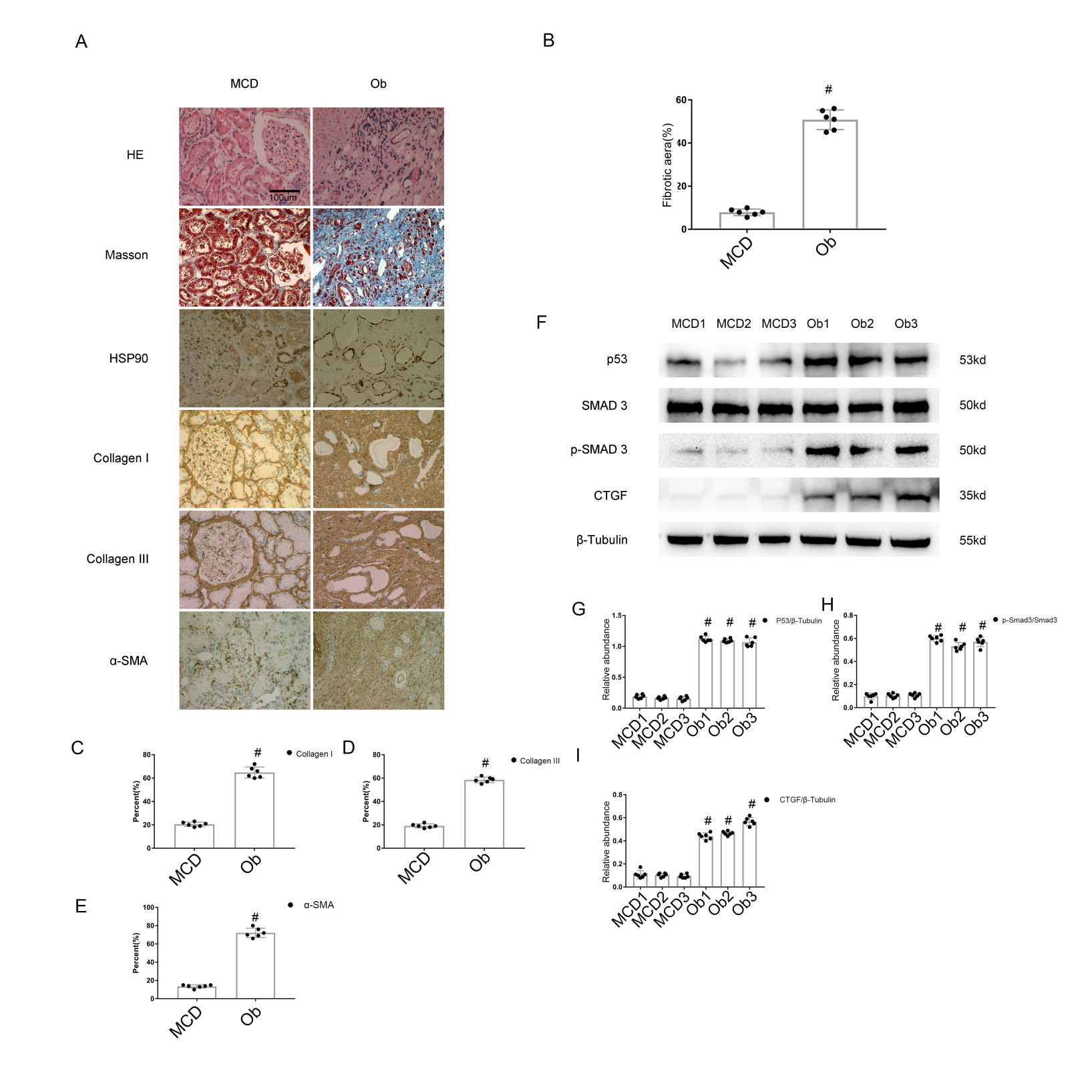


**Supplementary Figure 30**: **HSP90/Smad3 and p53/CTGF axis in patients with ON.** The samples of kidneys from MCD and Ob patients were collected. (A) The HE and Masson staining, and immunohistochemistry staining of HSP90, Col 1&III, and ɑ-SMA. (B) Quantification of the tubulointerstitial fibrosis. (C-E) Representative semi-quantitative immunostaining score of HSP90, Col 1&III, and ɑ-SMA. (F) Immunoblot of p53, smad3, p-smad3, CTGF,and β-tubulin. (G-I) Analysis of the grayscale image between them**.** These data are representative of at least four separate experiments shown as means±sd (n=6). # *P<0.05* versus MCD group. Each experiment(A,F) was repeated 6 times independently with similar results. (B-E,G-I) indicate the statistical Student's T test used(means ± sd,n=6,P<0.05). Original magnification, x400. Scar Bar:100um


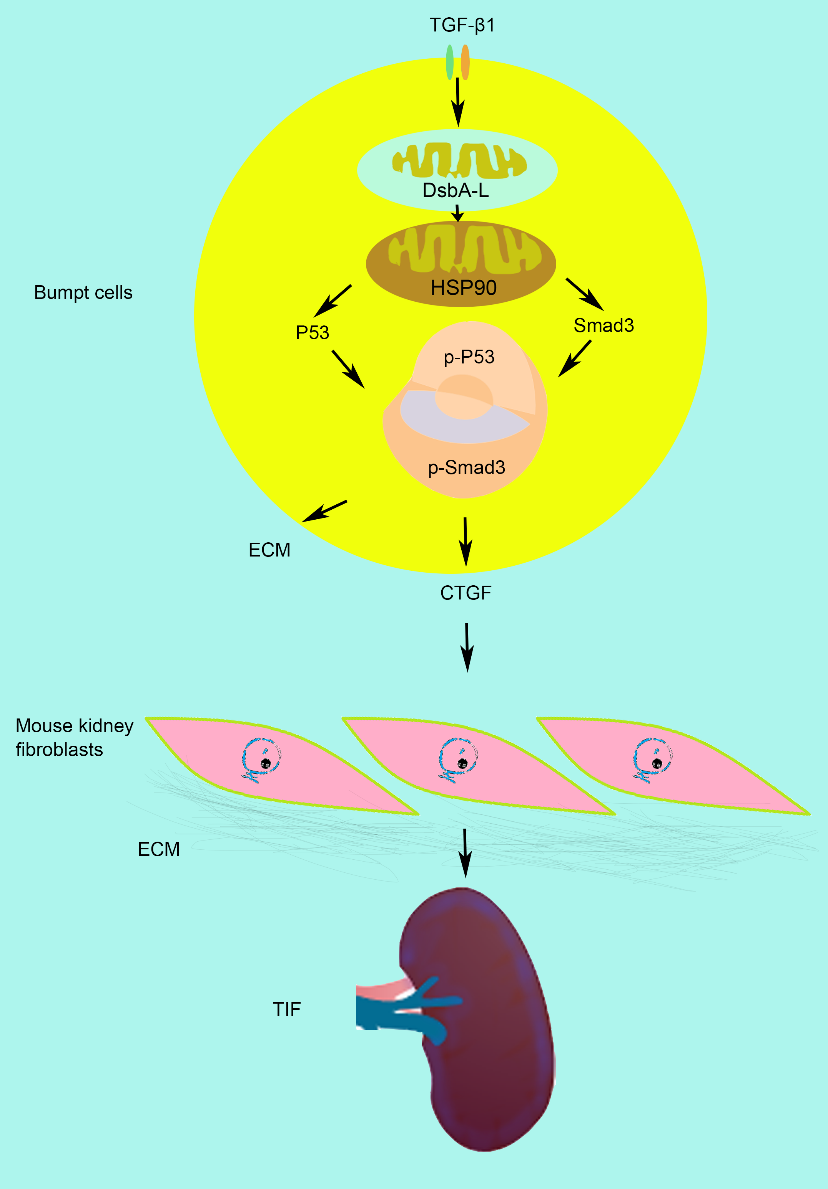


**Supplementary Figure 31: The role and molecular mechanism of tubular DsbA-L in UUO-induced renal fibrosis.** Tubular DsbA-L that caused by TGF-β1, interacted with HSP90 to activate the Smad3 and p53 signaling to lead to ECM accumulation and upregulate the CTGF expression in BUMPT cells, and CTGF subsequently stimulated fibroblasts to produce ECM **.**
