# Supplementary material for: DsbA-L mediated renal tubulointerstitial fibrosis in UUO mice
Source: Nat Commun. 2020 Sep 18;11:4467. doi: 10.1038/s41467-020-18304-z (PMC7501299; doi:10.1038/s41467-020-18304-z)
Supplement: Supplementary file 3 — Description of Additional Supplementary Files [file 41467_2020_18304_MOESM3_ESM.pdf]

Title: Supplementary Movie 1

Description: The movie dynamic showed the DsbA-L interacted with HSP90
