# Supplementary material for: DsbA-L mediated renal tubulointerstitial fibrosis in UUO mice
Source: Nat Commun. 2020 Sep 18;11:4467. doi: 10.1038/s41467-020-18304-z (PMC7501299; doi:10.1038/s41467-020-18304-z)
Supplement: Supplementary file 5 — Reporting Summary [file 41467_2020_18304_MOESM5_ESM.pdf]

nature research

Corresponding author(s): Dongshan zhang

Last updated by author(s): July-28-2020

Reporting Summary

Nature Research wishes to improve the reproducibility of the work that we publish. This form provides structure for consistency and transparency in reporting. For further information on Nature Research policies, see our Editorial Policies and the Editorial Policy Checklist.

Statistics

For all statistical analyses, confirm that the following items are present in the figure legend, table legend, main text, or Methods section.

n/a	Confirmed	
☐	☒	The exact sample size (*n*) for each experimental group/condition, given as a discrete number and unit of measurement
☐	☒	A statement on whether measurements were taken from distinct samples or whether the same sample was measured repeatedly
☐	☒	The statistical test(s) used AND whether they are one- or two-sided<br>*Only common tests should be described solely by name; describe more complex techniques in the Methods section.*
☒	☐	A description of all covariates tested
☐	☒	A description of any assumptions or corrections, such as tests of normality and adjustment for multiple comparisons
☐	☒	A full description of the statistical parameters including central tendency (e.g. means) or other basic estimates (e.g. regression coefficient) AND variation (e.g. standard deviation) or associated estimates of uncertainty (e.g. confidence intervals)
☐	☒	For null hypothesis testing, the test statistic (e.g. *F*, *t*, *r*) with confidence intervals, effect sizes, degrees of freedom and *P* value noted<br>*Give P values as exact values whenever suitable.*
☒	☐	For Bayesian analysis, information on the choice of priors and Markov chain Monte Carlo settings
☒	☐	For hierarchical and complex designs, identification of the appropriate level for tests and full reporting of outcomes
☒	☐	Estimates of effect sizes (e.g. Cohen's *d*, Pearson's *r*), indicating how they were calculated

Our web collection on statistics for biologists contains articles on many of the points above.

Software and code

Policy information about availability of computer code

Data collection	Olympus software,Zeiss Zen software were used to acquire images,LightCycler® 96 software
Data analysis	Graph Pad Prism 8.0, Adobe Photoshop CC 2018,Image-Pro Plus 6.0,ImageJ software.

For manuscripts utilizing custom algorithms or software that are central to the research but not yet described in published literature, software must be made available to editors and reviewers. We strongly encourage code deposition in a community repository (e.g. GitHub). See the Nature Research guidelines for submitting code & software for further information.

Data

Policy information about availability of data

All manuscripts must include a data availability statement. This statement should provide the following information, where applicable:

- Accession codes, unique identifiers, or web links for publicly available datasets
- A list of figures that have associated raw data
- A description of any restrictions on data availability

All data in this study are true and reliable.The data that support the findings of this study are available from the corresponding author upon reasonable request. The source data underlying Figs(1–2,4-6) and Supplementary Figs. (3–9、 11-15、 17-19、 21-22、 24-25、 27-30) are provided as a Source Data file. Source data are provided with this paper. The remaining figures data is uploaded as supplementary information.

Field-specific reporting

Please select the one below that is the best fit for your research. If you are not sure, read the appropriate sections before making your selection.

☒ Life sciences ☐ Behavioural & social sciences ☐ Ecological, evolutionary & environmental sciences

For a reference copy of the document with all sections, see nature.com/documents/nr-reporting-summary-flat.pdf

Life sciences study design

All studies must disclose on these points even when the disclosure is negative.

Sample size	Sample size was based on experience from previous published experiments leading to significant results.
Data exclusions	No data were excluded
Replication	Most experiments were replicated at least three times. All replications confirmed the findings.
Randomization	All animals are randomly selected for injection and modeling. All cell experiment groups are also randomly selected.
Blinding	Blinding test was done for scoring of histology.

Reporting for specific materials, systems and methods

We require information from authors about some types of materials, experimental systems and methods used in many studies. Here, indicate whether each material, system or method listed is relevant to your study. If you are not sure if a list item applies to your research, read the appropriate section before selecting a response.

Materials & experimental systems

n/a	Involved in the study	
☐	☒	Antibodies
☐	☒	Eukaryotic cell lines
☒	☐	Palaeontology and archaeology
☐	☒	Animals and other organisms
☐	☒	Human research participants
☒	☐	Clinical data
☒	☐	Dual use research of concern

Methods

n/a	Involved in the study	
☒	☐	ChIP-seq
☒	☐	Flow cytometry
☒	☐	MRI-based neuroimaging

Antibodies

Antibodies used	Anti-GAPDH ab181602,WB1:1000 , anti-HSP90 ab13492 ,WB1:1000 ,IF1:500, IP1:500 , anti-ß-actin ab8227WB1:1000 , anti-collagen # ab34710,WB1:1000,IHC:100 , anti-collagen III(ab7778WB1:1000,IHC1:100), anti-vimentin(ab92547), anti-"-SMA (ab32575)and anti-CTGF (ab6992) antibodies were obtained from Abcam (Cambridge Science Park,Cambridge, UK),Anti-p53(cst#2524),p-Smad3(#9520) ,Smad3 (#9523) and HA #3724 were purchased from Cell SignalingTechnology (Danvers, MA, USA), while Anti-COXIV(11242-1-AP,60251-1-lg 1:50 for IF) and Beta Tubulin (10068-1-AP)wereobtained from Proteintech (Rosemont, IL, USA). DsbA-L (WB 1:2000,IF1:500, IP1:500,IHC1:50)Anti-DsbA-L antibody was providedby Dr. Feng Liu Lab.
Validation	All antibodies have been verified by the official website.Anti-DsbA-L antibody has been verified in previous experiments.

Eukaryotic cell lines

Policy information about cell lines

Cell line source(s)	The mouse renal proximal tubular epithelial cell line BUMPT was obtained from Dr. Wilfred Lieberthal (Boston University School of Medicine).
Authentication	BUMPT cell lines have been unauthenticated.
Mycoplasma contamination	Cell lines were not tested for mycoplasma contamination but no indication of contamination was observed
Commonly misidentified lines (See ICLAC register)	No commonly misidentified cell lines were used

Animals and other organisms

Policy information about studies involving animals; ARRIVE guidelines recommended for reporting animal research

Laboratory animals	The proximal tubule-specific DabA-L-deletion mice were generated by crossing DsbA-L (flox/flox) mice (provided by Dr. Feng Liu Lab)with PEPCK-Cre mice (provided by Jackson Laboratory)
Wild animals	The study did not involve wild animals.
Field-collected samples	The study did not involve samples collected from the field
Ethics oversight	This animal experiment was approved by the Animal Ethics Committee of the Second Xiangya Hospital of Central South University.

Note that full information on the approval of the study protocol must also be provided in the manuscript.

Human research participants

Policy information about studies involving human research participants

Population characteristics	There is not a lot of demographic analysis, so it does not have characteristics, and all the samples are from the tissues removed by clinical surgery patients.
Recruitment	All organizations obtained the patient's consent and patients provided informed consent.Among them, Mcd tissue was collected from patients diagnosed with minimal change disease, and OB tissue is obstructive nephropathy tissue.
Ethics oversight	This experiment was approved by the Medical Ethics Committee of the Second Xiangya Hospital of Central South University. approval number is Lnc 2018-1. All organizations obtained the patient's consent. We declare all study complieswith all relevant ethical regulations for research with human participants and was carried out in compliance with the Declaration of Helsinki principles.

Note that full information on the approval of the study protocol must also be provided in the manuscript.

Clinical data

Policy information about clinical studies

All manuscripts should comply with the ICMJE guidelines for publication of clinical research and a completed CONSORT checklist must be included with all submissions.

Clinical trial registration	This study did not conduct clinical research experiments.
Study protocol	This study did not conduct clinical research experiments.
Data collection	This study did not conduct clinical research experiments.
Outcomes	This study did not conduct clinical research experiments.
